# Genetic and scRNA-seq Analysis Reveals Distinct Cell Populations that Contribute to Salivary Gland Development and Maintenance

**DOI:** 10.1038/s41598-018-32343-z

**Published:** 2018-09-19

**Authors:** Eun-Ah Christine Song, Sangwon Min, Akinsola Oyelakin, Kirsten Smalley, Jonathan E. Bard, Lan Liao, Jianming Xu, Rose-Anne Romano

**Affiliations:** 1 Department of Oral Biology, School of Dental Medicine, State University of New York at Buffalo, Buffalo, New York 14214 USA; 2 Department of Biochemistry, Jacobs School of Medicine and Biomedical Sciences, State University of New York at Buffalo, Buffalo, New York 14203 USA; 3 Genomics and Bioinformatics Core, State University of New York at Buffalo, Buffalo, New York 14222 USA; 4 Department of Molecular and Cellular Biology, Baylor College of Medicine, Houston, Texas 77030 USA

## Abstract

Stem and progenitor cells of the submandibular salivary gland (SMG) give rise to, maintain, and regenerate the multiple lineages of mature epithelial cells including those belonging to the ductal, acinar, basal and myoepithelial subtypes. Here we have exploited single cell RNA-sequencing and *in vivo* genetic lineage tracing technologies to generate a detailed map of the cell fate trajectories and branch points of the basal and myoepithelial cell populations of the mouse SMG during embryonic development and in adults. Our studies show that the transcription factor p63 and alpha-smooth muscle actin (SMA) serve as faithful markers of the basal and myoepithelial cell lineages, respectively and that both cell types are endowed with progenitor cell properties. However, p63^+^ basal and SMA^+^ myoepithelial cells exhibit distinct cell fates by virtue of maintaining different cellular lineages during morphogenesis and in adults. Collectively, our results reveal the dynamic and complex nature of the diverse SMG cell populations and highlight the distinct differentiation potential of the p63 and SMA expressing subtypes in the stem and progenitor cell hierarchy. Long term these findings have profound implications towards a better understanding of the molecular mechanisms that dictate lineage commitment and differentiation programs during development and adult gland maintenance.

## Introduction

Salivary gland (SG) morphogenesis is highly dependent on distinct populations of epithelial stem and progenitor cells that undergo a number of dynamic cellular processes including fate specification, lineage commitment and differentiation to generate the diverse cell lineages that make up this gland. In adults, the delicate balance between proliferation and differentiation of epithelial stem/progenitor cells must be tightly regulated in order to maintain and regenerate the mature cell lineages that sustain SG function. The SG is comprised of several epithelial cell types including acinar, ductal, basal and myoepithelial cells which are surrounded by a dynamic extracellular matrix^1^. The main secretory units of the salivary gland are the acini, which are designated as either serous or mucous depending on the consistency of their secretions. Serous acinar cells produce watery, protein rich secretions, while mucous acinar cells generate viscous secretions, which are largely made up of mucins^2^. Once produced, saliva is then secreted into the lumens of the ducts, where the ionic composition of the saliva is modified before it travels to the oral cavity through an intricate and interconnected ductal network^3^. Surrounding the acini and interspersed within the cells of the basal layer, are a specialized cell type referred to as myoepithelial cells^4^.

In mice, SG morphogenesis begins during early embryonic development. The rudimentary salivary gland is first visible as a thickening of the adjoining oral epithelium which occurs at approximately embryonic day 11.5 (E11.5), commonly known as the Prebud stage^1,5,6^. During the subsequent Initial Bud stage (E12.5), the thickened epithelium invaginates into the underlying mesenchyme thus forming a primary bud which will serve as the precursor of the main duct of the salivary gland. The gland continues to mature and at E14.5, it commences a program of branching morphogenesis to generate the intricate ductal network that will be required for channeling the saliva into the oral cavity. This Pseudoglandular stage also marks the formation of the acini, which are the main secretory units of the salivary gland. At the Canalicular stage (E16), the gland is highly branched with lumenization of the main secretory duct nearing completion^1,7^. The onset of cytodifferentiation also occurs at this stage, a process which continues until birth. During the final stages of morphogenesis, the Terminal Bud stage (E18), expansion of the acini and lumenization of both the ducts and acini nears completion resulting in a continuous ductal network connecting the acini to the oral cavity^8,9^. After birth, acini maturation and differentiation continue, and by puberty, differentiation of the granular convoluted tubules is completed^1,7^.

Given the critical importance of stem/progenitor cells in normal SG development, it is essential to define their cell fate potentials, and in particular to ascertain where and how such choices are specified over the course of development. Such information is not only valuable for identifying regulatory networks and pathways that are important in directing cell fate decisions, but also critical for informing on regulatory programs crucial for gland growth, maintenance and regeneration. Over the last several years the use of genetic lineage tracing technologies to map the fate and progeny of stem/progenitor cells in the salivary gland have begun to shed light on the dynamics of cell fate specification patterns during development, tissue maintenance and repair. Studies examining early cell fate specification programs have identified distinct classes of stem/progenitor cells which give rise to the various epithelial cell lineages of the salivary gland. Lineage tracing experiments investigating the contribution of the Sex-determining Region Y (SRY) box (Sox) family members Sox2 and Sox9, have demonstrated that both Sox2-positive (Sox2^+^) and Sox9^+^ cells contribute to the acinar and ductal cell lineages during development^10,11^. In adult glands however, Sox2^+^ cells remained lineage restricted and maintained a subpopulation of acinar cells^11^. Moreover, Achaete-Scute family BHLH transcription factor 3 (Ascl3) cells have also been shown to mark a stem/progenitor cell population capable of contributing to both acinar and ductal cell lineages^12^. Interestingly, a recent genetic lineage tracing study using the acinar cell specific gene driver, Mist1 reported that adult acinar cells are maintained through acinar self-duplication indicating that acinar cells are capable of giving rise to more acinar cells^13^.

While prior studies have identified stem/progenitor cell populations that maintain the ductal and acinar cell lineages, similar detailed analyses examining the basal and myoepithelial cell populations is wanting. Lineage tracing experiments using keratin 14 (K14) and K5, two broadly expressed markers of basal and myoepithelial cells have demonstrated that these cells function as multipotent stem/progenitor cells as their progeny contribute to all epithelial cell lineages during development^14,15^. In adults however, K14^+^ cells remained lineage restricted and maintained the differentiated cells of the ducts^16,17^. Given the likely heterogeneity of the basal and myoepithelial cells, additional studies focusing on other defined markers within these cellular populations may provide further insight into the stem/progenitor cell potentials and cell-lineage dynamics.

In this study, we integrate single-cell RNA sequencing (scRNA-seq) with *in vivo* genetic lineage tracing experiments in the mouse submandibular gland (SMG) to map the fate of the basal and myoepithelial cells. By utilizing the transcription factor p63, a well-established stem/progenitor cell marker which is highly expressed in the basal and myoepithelial cell compartments, we demonstrate that p63^+^ cells function as multipotent progenitors that give rise to all epithelial cells lineages during development and in adult gland maintenance. To probe the stem/progenitor cell potentials of the myoepithelial cells (MECs), we selectively labelled these cells by taking advantage of the restricted expression pattern of alpha-smooth muscle actin (SMA). We find that during embryogenesis, SMA-positive MECs are lineage restricted and contribute to the basal and myoepithelial cell lineages. Conversely, lineage tracing experiments performed in adult animals reveal that MECs contribute to both the myoepithelial and ductal cell lineages. Overall, our studies have identified new players in the stem/progenitor cell hierarchy in the salivary gland and provide novel insights into the dynamics of these important cell populations during both development and adult gland maintenance. Long-term, these studies have broad implications in the development of cell-based therapies to restore the function of damaged salivary glands.

## Results

### Single cell transcriptome analysis of the mouse submandibular salivary gland

To gain a more detailed understanding of the molecular and cellular heterogeneity of the various cell types within the SMG, we performed single-cell RNA-sequencing (scRNA-seq) analysis. Single-cell RNA-seq was carried out on submandibular glands of postnatal day 8 (P8) mice, a time point in which the SG has completed morphogenesis and all cell lineages have been shown to be established. SMG were isolated, dissociated into single cell suspensions and subjected to scRNA-seq using the 10X Genomics sequencing platform for single-cell capture to achieve in-depth expression profiling of individual salivary gland epithelial cells. Sequencing data from a total of 1,013 cells was subject to further analysis after filtering based on various quality control metrics yielded a mean of 2,241 genes and 301,852 unique reads per cell.

**Figure 1:**
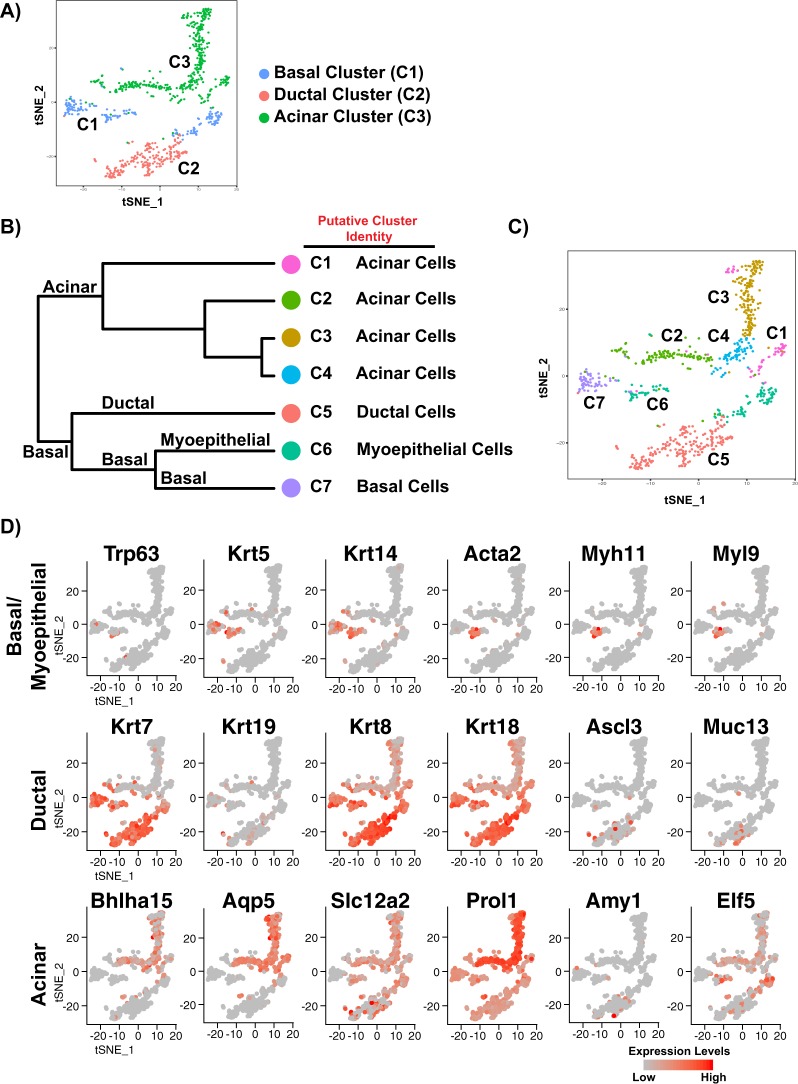
Identification of salivary gland epithelial cell clusters by single-cell RNA sequencing. (**A**) Salivary gland epithelial cell transcriptomes visualized with t-distributed stochastic neighbor embedded (t-SNE), colored according to unsupervised clustering. (**B**) Hierarchical clustering analysis based on the log-transformed mean expression values of the 7 clusters. The tree was computed based on Spearman’s rank correlation with Ward linkage. (**C**) t-SNE plot based on the hierarchical clustering analysis performed in panel B above. (**D**) Epithelial cell clusters were interrogated for the expression of known basal, myoepithelial, ductal and acinar genes to determine their patterning amongst the cell clusters. C- cluster.

To investigate the level of molecular heterogeneity, we performed unsupervised clustering with affinity propagation based on the expression of high variance genes. Importantly, all clusters were generated in an unbiased manner without cross-referencing published gene expression data sets. Interestingly, our initial analyses identified 3 major clusters (representing distinct groups of cells) which were visualized via t-distributed stochastic neighbor embedding (t-SNE)^18^ (Fig. 1A). Examination of the expression of known and/or validated markers for the mature SMG cell lineages including basal cell markers (*Trp63*, *Krt5* and *Krt14*), ductal cell markers (*Krt7* and *Krt8*), and acinar markers (*Bhlha15* and *Aqp5*) allowed us to assign the 3 major clusters (C) to the basal (C1), ductal (C2) and acinar cell lineages (C3) (Fig. 1A).

Next, we applied hierarchical clustering to each of the identified groups to further resolve the extent of cellular heterogeneity and to identify potential subgroups of cell-populations that are distinct and possess unique gene expression profiles. This resulted in a total of 7 distinct SMG epithelial cell clusters. To further characterize the clusters, we identified differentially expressed genes and inferred putative identities based on known markers (Fig. 1B,C and Supplementary Table S1). As demonstrated in Fig. 1B, multiple clusters of cells were assigned to the same putative cell type. In the acinar lineage, we found 4 subgroups (C1-C4) which showed tell-tale characteristics of acinar cells such as high expression of the transcription factor *Bhlha15* (also known as Mist1) and additional markers including *Aqp5, Prol1* (also known as Muc10) and *Pip* (Fig. 1B–D)^9,13,19,20^. Within the ductal compartment, we identified the C5 subtype, which showed enrichment of known markers associated with the ductal lineage; *Krt7*, *Krt8*, and *Krt18*^14,21,22^. Finally, within the basal lineage we found 2 subgroups, highlighting the heterogeneity in this cell compartment. Cells segregating to the C7 cluster showed defined characteristics of basal cells including expression of the genes *Trp63*, *Krt5* and *Krt14* while cells from C6 were enriched for expression of myoepithelial specific genes such as *Acta2* (SMA), *Myh11* and *Myl9* in addition to the core basal genes (Fig. 1B–D)^23^. Interestingly, a closer examination of C6 revealed that cells within this cluster also expressed ductal genes such as *Krt7*, *Krt8*, and *Krt18*, as well as acinar specific genes *Prol1* and *Elf5*^9,24^ (Fig. 1D). It is plausible that this cluster may represent a mixed-lineage, or a mixed population of cells that may be poised for commitment to the different cell lineages similar to mixed cellular subsets that have been reported in the pancreas, intestines, and mammary glands^23,25–27^. Taken together, our scRNA-seq analyses identified 3 major cell clusters representing the basal, ductal and acinar cell lineages, highlighting the degree of cellular heterogeneity within the salivary gland.

### Temporal and spatial expression analysis of the basal and myoepithelial cell markers p63 and alpha-smooth muscle actin (SMA) during mouse salivary gland development and in adults

Our scRNA-seq results revealed two subpopulations of basal cells. Cluster C7, which represented the basal cells, showed enriched gene expression levels of *Trp63*, while cluster C6 displayed restricted gene expression levels of *Acta2*. The lineage specific transcription factor *Trp63*, specifically the ΔNp63 isoform, is highly expressed in the basal and myoepithelial cells of glandular tissues such as those of the mammary, lacrimal and salivary glands where it plays important roles in stem cell self-renewal, morphogenesis and directing differentiation programs^28–34^. In addition to the keratin and basal cell markers K5, K14, and ΔNp63, myoepithelial cells exclusively express the myofilament protein alpha-smooth muscle actin (SMA)^35^. Using p63 and SMA as markers for basal and myoepithelial cells respectively, we sought to determine when these cell types were established during salivary gland morphogenesis. To address this, we first investigated the temporal and spatial expression patterns of p63 and SMA during embryonic submandibular salivary development and in adult mice. We began our analysis by examining the mRNA expression levels of the *Trp63* gene. By utilizing RNA-sequencing (RNA-seq) datasets of the mouse submandibular gland generated by our laboratory, we found that of the various *Trp63* isoforms, including *TAp63* and *ΔNp63*, *ΔNp63* were the only isoforms expressed in the submandibular salivary gland (Supplementary Fig. S1A)^36^. Indeed, *ΔNp63* mRNA transcripts were detected as early as embryonic day 14.5 (E14.5) and gradually decreased during morphogenesis and remained low in the adult glands (Supplementary Fig. S1B). We similarly mined our RNA-seq datasets to determine when the *Acta2* gene, which encodes for SMA protein, was expressed during salivary gland morphogenesis. Our analysis revealed that *Acta2* mRNA was detected as early as E14.5 and steadily increased during morphogenesis and remained elevated in adult glands (Supplementary Fig. S1C)^36^.

**Figure 2:**
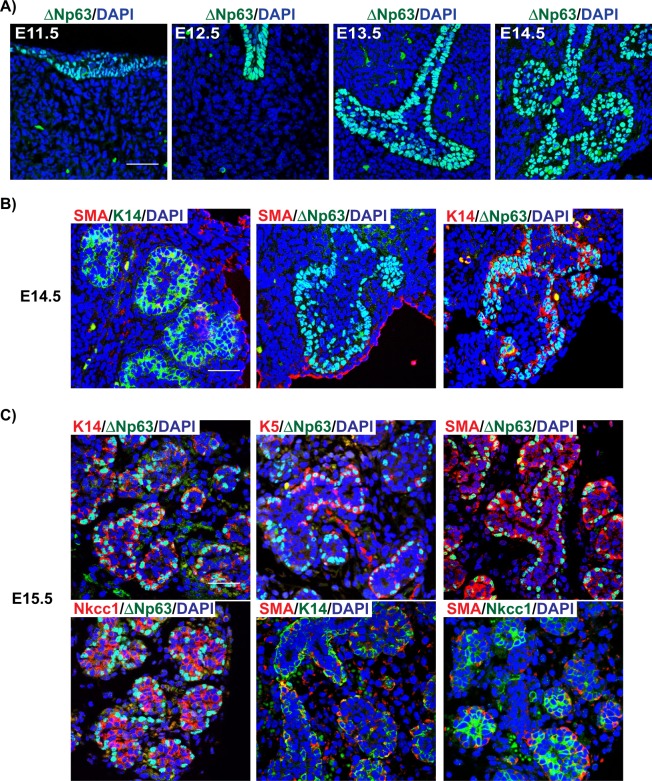
Spatial expression pattern of ΔNp63 and SMA proteins during the early stages of salivary gland morphogenesis. (**A**) ΔNp63 is expressed in the epithelial cells of the placode as well as the developing buds and end buds during the early stages of salivary gland development. (**B**) Expression of the basal and myoepithelial cell markers SMA, K14 and ΔNp63 at E14.5. (**C**) At E15.5 ΔNp63 and SMA expression is observed in the basal and myoepithelial cells surrounding the developing ducts and acini. Scale bar 37 μm.

Armed with this information we next examined the protein expression patterns of ΔNp63 and SMA by immunofluorescence studies of the SMG. Given that the earliest RNA-seq datasets available were limited to E14.5, we began our protein expression analysis to include the initial stages of gland morphogenesis. Using ΔNp63 specific antibodies, we found that at the gland initiation stage (E11.5), ΔNp63 was expressed in all the epithelial cells of the developing placode (Fig. 2A). From the Initial Bud (E12.5) to the Pseudoglandular stage (E14.5), ΔNp63 expression was observed in the developing buds and end buds (Fig. 2A,B). By E15.5, ΔNp63 was co-expressed with the progenitor cell markers K5 and K14 in the outer layer of the developing ducts (Fig. 2C)^14,15^. During this stage, ΔNp63 expression was also detected in the myoepithelial cells surrounding the developing acini as shown by co-staining of ΔNp63 with SMA^4,32^ and the acinar cell marker Na^+^/K^+^/2Cl^−^ co-transporter (Nkcc1) (Fig. 2C). Interestingly, by the later stages of embryonic development (E18.5), ΔNp63 expression was not restricted to the K5 or K14 cellular populations. Indeed, quantification of ΔNp63^+^, K5^+^, and K14^+^ cells at E18.5 revealed distinct ΔNp63^+^ cell populations suggesting that these three populations may have different progenitor cell properties (Supplementary Fig. S2). As expected, TAp63 protein was not detected at any of the time points examined, further confirming our RNA-seq results (data not shown and Supplementary Fig. S1A).

**Figure 3:**
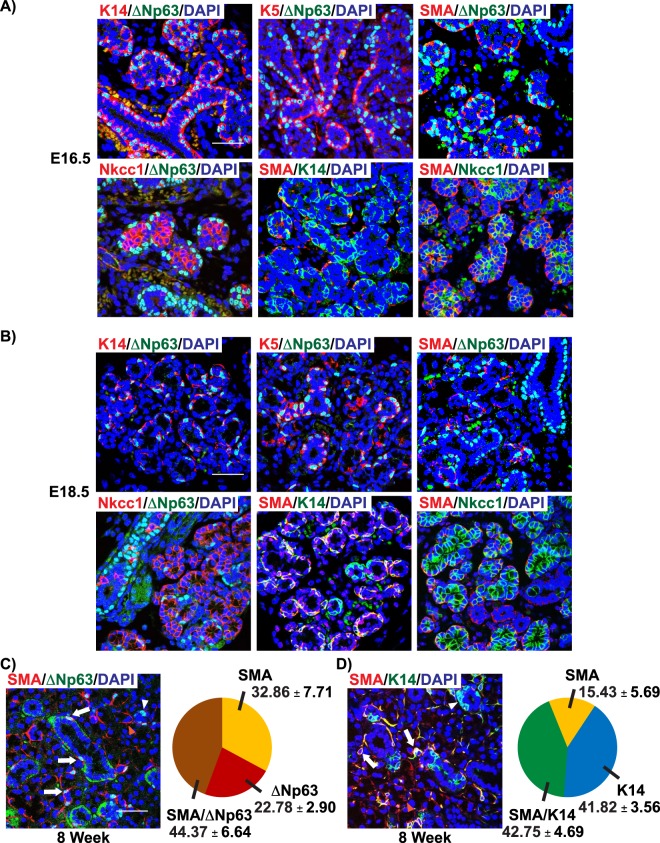
Expression pattern of ΔNp63 and SMA during the later stages of salivary gland development and adults. (**A**,**B**) Expression of ΔNp63 and SMA at E16.5 and E18.5. (**C**) Expression and quantification analyses of SMA and ΔNp63 in 8-week old adult glands. Arrow- SMA^+^/∆Np63^+^ cells, white arrowhead- SMA^−^/∆Np63^+^ cells, orange arrowhead-SMA^+^/∆Np63^−^ cells. (**D**) Expression and quantification analyses of SMA and K14 in 8-week old adult glands. Arrow- SMA^+^/K14^+^ cells, white arrowhead- SMA^−^/K14^+^ cells, orange arrowhead-SMA^+^/K14^−^ cells. Data are represented as mean ± standard deviation (S.D.). Scale bar 37 μm. *n* = *3*.

While our RNA-seq analysis of *Acta2* mRNA revealed expression at E14.5, we were unable to detect SMA protein expression levels at this developmental time point - in good agreement with previous reports (Fig. 2B left two panels)^9,37^. However, by E15.5 SMA^+^ myoepithelial cells were detected surrounding the developing ducts, as shown by co-staining with ΔNp63 and K14, suggesting that the myoepithelial cell lineage is established as early as E14.5, a stage before the protein expression levels of SMA could be detected (Fig. 2B,C). Moreover, co-localization of SMA with both ΔNp63 and K14 was maintained over the course of embryonic development, suggesting that myoepithelial cells are established from ΔNp63^+^/K14^+^ cells (Figs 2B,C and 3A,B). SMA^+^ myoepithelial cells were also located around the developing acini as demonstrated by co-staining with Nkcc1 (Figs 2C and 3A,B).

Our embryonic expression analysis of SMA, K5, K14, and ΔNp63, prompted us to investigate the expression pattern of these markers in 8-week adult submandibular glands. Co-staining of SMA and ∆Np63 revealed a large number of ∆Np63^+^/SMA^+^ double positive cells surrounding both the acini and the ducts (Fig. 3C, white arrow). Interestingly, we identified additional distinct cellular sub-populations which included ∆Np63^+^/SMA^−^ cells surrounding the ducts, and ∆Np63^−^/SMA^+^ MECs surrounding the acini (Fig. 3C, white arrowhead and orange arrowhead respectively). These observations pointed to the possibility of a unique SMA^+^ cellular population of MECs at this age, something which we did not observe during embryogenesis. Indeed, our quantification analyses confirmed the existence of a unique ∆Np63^−^/SMA^+^ cellular population representing approximately 32.86% ± 7.71% of the total number of ∆Np63^+^ and SMA^+^ cells (Fig. 3C). This was further confirmed by quantification of SMA^+^ and K14^+^ populations which revealed a similarly unique K14^−^/SMA^+^ cell population of ~15.43% ± 5.69% (Fig. 3D). We also examined the status of the ∆Np63/K14 and ∆Np63/K5 cell populations and found that of these cells, 2.52% ± 0.49% were ∆Np63^+^/K14^−^ while 7.01% ± 1.08% were ∆Np63^+^/K5^−^, suggesting that similar to the SMA-restricted cell types, there exists a unique population of ∆Np63^+^ basal cells within the gland (Supplementary Fig. S4A,B). Overall, our results demonstrate that during the early stages of development, ∆Np63 cells are located within the developing placode and over the later stages of morphogenesis, ∆Np63 expression is restricted to the basal cells surrounding the ducts and the SMA^+^ myoepithelial cells surrounding the acini (Supplementary Fig. S3). Moreover, our results suggest that although SMA expressing myoepithelial cells are first detected at E15.5, it is likely that this cell lineage is established earlier and presumably arise from the ∆Np63^+^/K14^+^ basal cells. Finally, our quantification analysis of the various subpopulations of basal and myoepithelial cells in adult glands revealed unique sub-populations of ∆Np63^+^ and SMA^+^ cells.

### Contribution of p63^+^ and SMA^+^ cell populations during salivary gland morphogenesis

**Figure 4:**
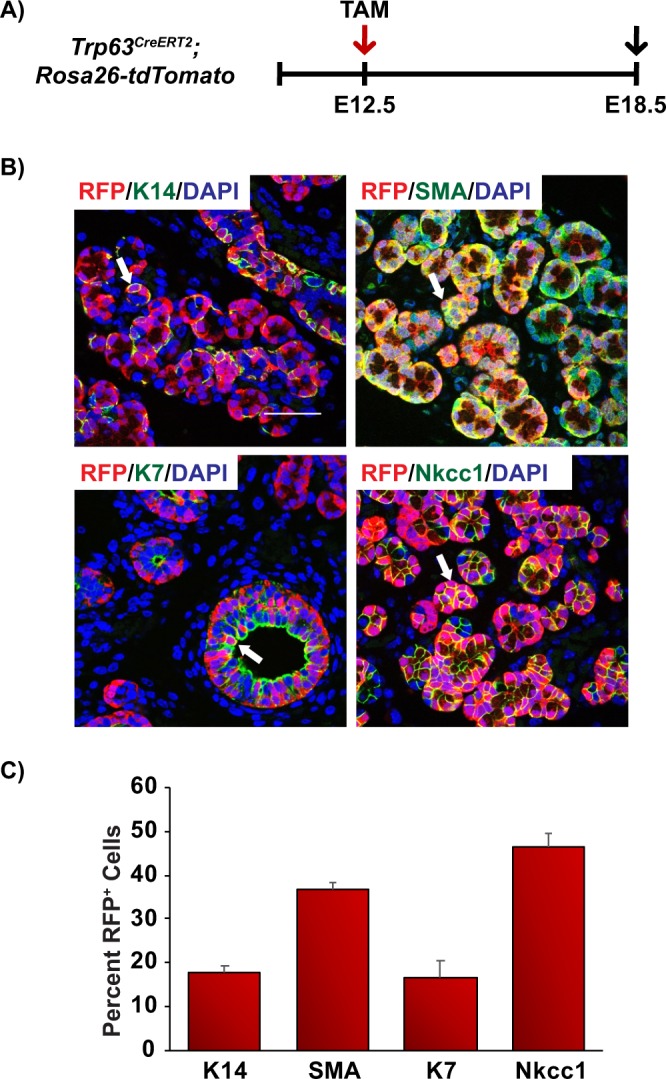
Contribution of p63^+^ cells during salivary gland morphogenesis. (**A**) Schematic of the experimental timeline used for the embryonic genetic lineage tracing experiments in the *Trp63*^*CreERT2*^*;Rosa26-tdTomato* mice. RFP expression was induced at E12.5 by injection of TAM to pregnant females and cells were traced for 6 days and glands were dissected at E18.5. (**B**) p63^+^ cells contribute to all epithelial cell lineages during submandibular gland morphogenesis. (**C**) Quantification of the percentage of RFP^+^ cells which co-express the various cell lineage markers as indicated. Data are represented as the mean ± standard deviation (S.D.). Arrows highlight double positive cells as indicated. Scale bar 37 μm. TAM- tamoxifen, E-embryonic. *n* = *4*.

To better understand the stem/progenitor cell trajectories and define the branch point of the basal and myoepithelial cell lineages, we performed genetic lineage tracing analysis to trace the progeny of both p63^+^ and SMA^+^ cells during embryogenesis and in adult glands. To characterize the stem/progenitor cell potential of p63^+^ epithelial cells in submandibular gland development, mice expressing a Tamoxifen (TAM) inducible Cre recombinase fused to the estrogen-ligand binding domain ERT2 under the control of the *Trp63* gene locus (*Trp63*^*CreERT2*^) were crossed with the *Rosa26-tdTomato* red fluorescent protein (RFP) reporter mouse strain (*Trp63*^*CreERT2*^*;Rosa26-tdTomato*)^38^. This strain carries a loxP STOP cassette upstream of the RFP variant (tdTomato) under the control of the ubiquitously active *Rosa26* locus. In the presence of active Cre recombinase, the STOP cassette is excised through recombination, resulting in irreversible RFP expression. To assess the contribution of p63-expressing cells to submandibular gland development, TAM was administered to pregnant females at E12.5 and embryonic glands were examined for RFP expression at E18.5 (Fig. 4A). Our analysis revealed co-localization of RFP^+^ cells to all the epithelial cells of the gland including the K14^+^ basal, SMA^+^ myoepithelial, K7^+^ luminal, and Nkcc1^+^ acinar cells^39^ (Fig. 4B). Quantification of the percentage of RFP^+^ cells which co-express the various cell lineage markers is shown in Fig. 4C. Importantly, no staining was observed in submandibular glands of control littermates (data not shown) demonstrating the specificity of the model system. Moreover, co-staining of glands with RFP and ΔNp63 revealed faithful expression of RFP to ΔNp63^+^ cells, consistent with published reports (Supplementary Fig. S5A)^38^.

**Figure 5:**
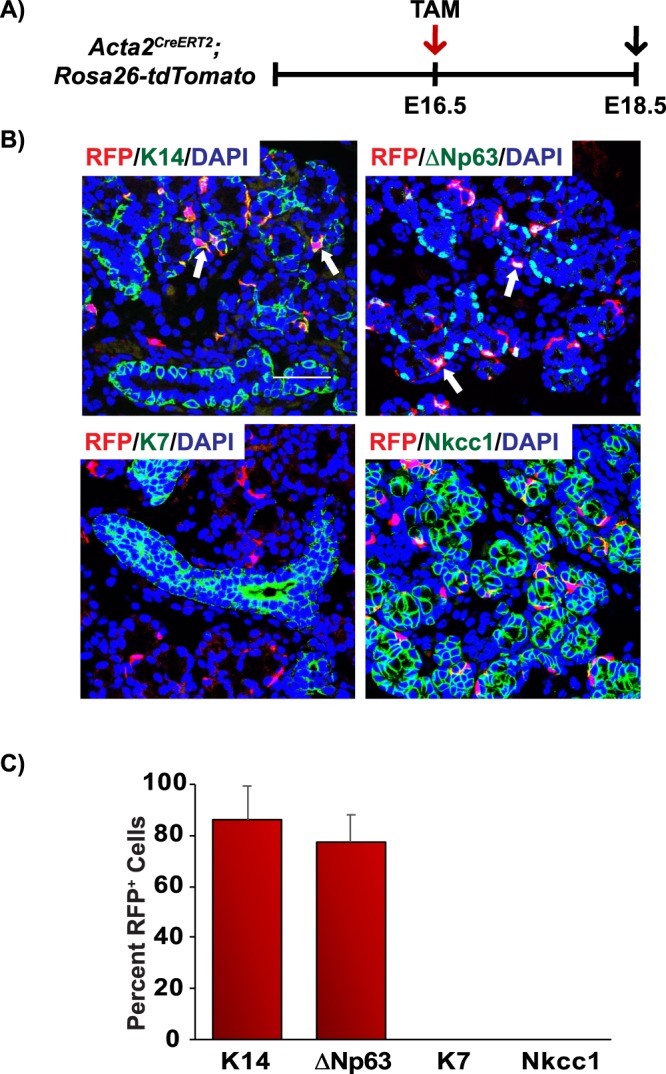
Contribution of SMA^+^ cells during salivary gland development. (**A**) Schematic depicting the experimental timeline for the genetic lineage tracing studies performed in the *Acta2*^*CreERT2*^*;Rosa26-tdTomato* mice. (**B**) SMA^+^ cells contribute exclusively to the basal and myoepithelial cell lineages. (**C**) Quantification of the percentage of RFP^+^ cells which co-express the various cell lineage markers as indicated. Data are represented as mean ± standard deviation (S.D.). Arrows highlight double positive cells as indicated. Scale bar 37 μm. TAM- tamoxifen, E-embryonic. *n* = *4*.

To complement these observations, we assessed the contribution of the SMA^+^ myoepithelial cells to submandibular gland morphogenesis by crossing *Acta2*^*CreERT2*^ expressing animals with the *Rosa26-tdTomato* reporter mouse strain (*Acta2*^*CreERT2*^*;Rosa26-tdTomato*)^40^. TAM was administered at E16.5 and glands were examined for RFP expression at E18.5 (Fig. 5A). While no RFP expression was observed in the acinar cells, as demonstrated by the absence of RFP and Nkcc1 co-staining, our analysis revealed co-localization of RFP positive cells to the K14^+^ and ∆Np63^+^ basal cells (Fig. 5B). In order to confirm the contribution of SMA^+^ cells to the basal cell lineage we performed triple immunofluorescence staining to examine the expression profile of RFP, K14 and SMA in these glands (Supplementary Fig. S6). We reasoned that cells which co-expressed RFP^+^/K14^+^/SMA^+^ represented SMA^+^ derived myoepithelial cells, while RFP^+^/K14^+^/SMA^−^ cells represented SMA^+^ derived K14^+^ basal cells and not myoepithelial cells, as these cells are SMA^−^. Indeed, quantification of the percent RFP^+^, SMA^+^, K14^+^ cells revealed that while a majority of the cells were RFP^+^/SMA^+^/K14^+^ (98.75 ± 0.36%) a small percentage (1.24 ± 0.36%) were RFP^+^/K14^+^/SMA^−^ suggesting that SMA^+^ cells contribute to the K14^+^ basal cell lineage (Supplementary Fig. S6A). Moreover, we did not observe any detectable RFP expression in the ductal cells as demonstrated by co-staining with the ductal marker K7 (Fig. 5B). Quantification of our results are found in Fig. 5C and Supplementary Fig. S6A. The expression of RFP was specific since no staining was observed in submandibular glands of control littermates (data not shown). In addition, co-staining of glands with RFP and SMA revealed complete overlap, confirming the faithful expression of RFP to the myoepithelial cells, consistent with previous reports (Supplementary Fig. S5B)^41,42^. Overall, the results from the lineage tracing experiments performed during embryogenesis demonstrated that p63^+^ cells contributed to all cell lineages during development. In contrast, SMA^+^ cells were restricted in their potential and gave rise to the cell populations of the basal and myoepithelial lineages.

### p63^+^ and SMA^+^ stem/progenitor cells maintain different mature cell lineages in adult gland maintenance

**Figure 6:**
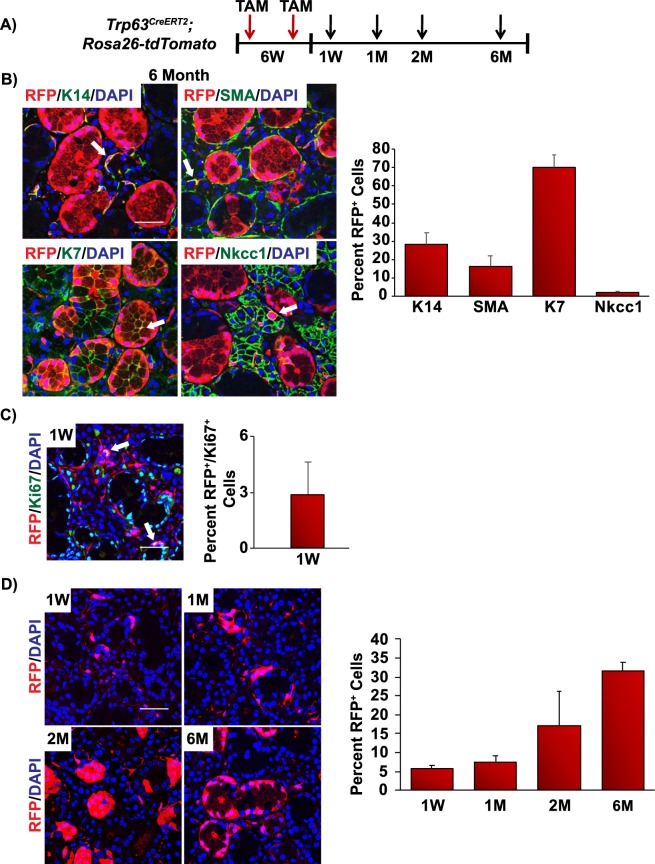
p63^+^ cells maintain the mature cell lineages of the submandibular gland. (**A**) Experimental timeline used for the genetic lineage tracing experiments in adult *Trp63*^*CreERT2*^*;Rosa26-tdTomato* mice. RFP expression was induced by TAM injection in 6-week old adult mice and cells were traced for 1 week and 1, 2 and 6 months. (**B**) RFP expression was detected in all the epithelial cell lineages which comprise the salivary gland in *Trp63*^*CreERT2*^*;Rosa26-tdTomato* mice at 6 months (left panel). Quantification of the percentage of RFP^+^ cells which co-express the various cell lineage markers as indicated (right panel). (**C**) Co-staining of RFP and the proliferative marker Ki67 in adult submandibular glands 1 week after TAM administration (left panel). Quantification of RFP^+^/Ki67^+^ cells in submandibular glands, expressed as a percentage of total single Ki67^+^ cells (right panel). (**D**) Submandibular glands were evaluated for RFP expression 1 week and 1, 2 and 6 months following TAM administration (left panel). Quantification of RFP^+^ cells per total number of cells (nuclei) counted in glands isolated at the indicated time points after TAM administration (right panel). Data are represented as mean ± standard deviation (S.D.). Arrows highlight double positive cells as indicated. W- week, M- month. Scale bar 37 μm. *n* = *4*.

To better understand the role p63^+^ stem/progenitor cells play in adult gland maintenance, TAM was administered to 6-week adult *Trp63*^*CreERT2*^*;Rosa26-tdTomato* (p63 bi-genic) mice and RFP expression was assessed after 6 months (Fig. 6A). As expected, RFP^+^ cells co-localized to both the K14^+^ basal and SMA^+^ myoepithelial cells (Fig. 6B). Indeed, RFP^+^/K14^+^ cells and RFP^+^/SMA^+^ cells were readily detected as early as 1-week after TAM administration (Supplementary Fig. S7B). Interestingly, co-staining of RFP with the ductal marker K7 revealed localization of RFP^+^ cells to the luminal cells of the ducts (Fig. 6B, arrow). To determine if p63^+^ derived cells contributed to the acinar cell lineage we examined for expression of RFP^+^ cells in the Nkcc1^+^ acinar expressing cells. While we were unable to detect any RFP^+^/Nkcc1^+^ double positive acinar cells after 2 months of TAM administration, we observed a modest number of RFP^+^/Nkcc1^+^ cells 6 months after RFP induction (Supplementary Fig. S7B and Fig. 6B- arrow, respectively). These findings were confirmed by co-staining of the TAM-treated 6 month glands from both male and female animals with two additional acinar specific markers; Aqp5 and Mist1 (Supplementary Fig. S8). Quantification of the percentage of RFP^+^ cells which co-express the various cell lineage markers is shown in Fig. 6B and Supplementary Fig. S8.

To delve further into the dynamics of the p63^+^ cells of the submandibular gland, we assessed the proliferative properties of these cells. Six-week old adult mice were administered TAM followed by a 1 week chase. Submandibular glands were harvested and analyzed for the expression status of Ki67, a marker of actively dividing cells^43^ and RFP. Upon quantification of the total number of Ki67^+^ cells, we found that almost 3% were RFP^+^/Ki67^+^ double positive and in active phases of the cell cycle, confirming active proliferation of these cells in adult submandibular glands (Fig. 6C). Interestingly, quantification of RFP expression 1 week, 1, 2 and 6 months post induction revealed an approximate 6-fold increase in the number of RFP^+^ cells (Fig. 6D). The persistence of RFP positive cells is highly suggestive of progenitor and/or stem cell activity of p63^+^ basal cells, similar to what has been reported in other epithelial rich organs^34,38,44–46^.

**Figure 7:**
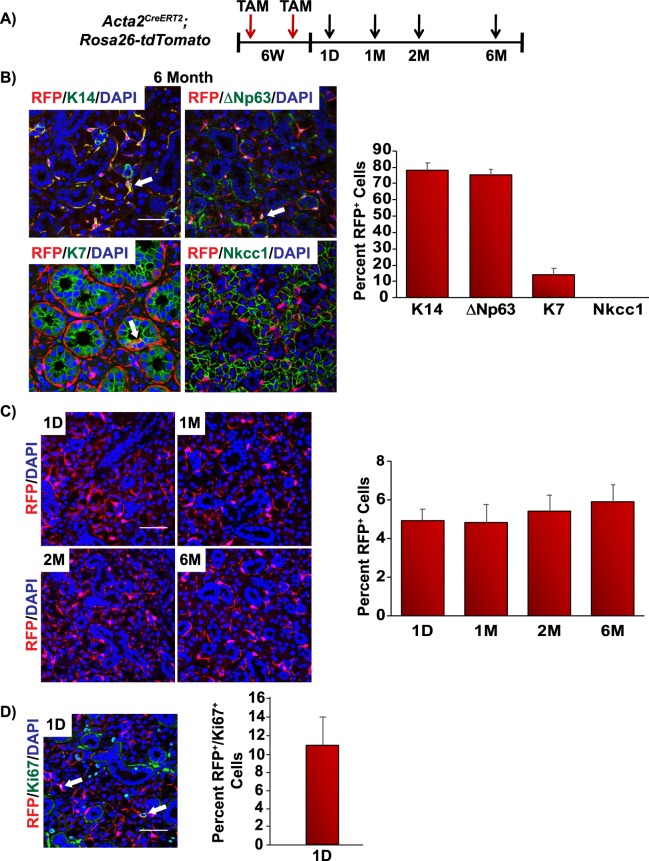
SMA^+^ cells maintain the myoepithelial and ductal cell lineages in the adult submandibular gland. (**A**) Experimental timeline used for the genetic lineage tracing experiments in adult *Acta2*^*CreERT2*^*;Rosa26-tdTomato* mice. RFP expression was induced by TAM injection in 6-week old adult mice and cells were traced for 1 day, 1, 2 and 6 months. (**B**) RFP expression was detected in the myoepithelial and ductal cell lineages (left panel). Quantification of the percentage of RFP^+^ cells which co-express the various cell lineage markers as indicated (right panel). (**C**) Submandibular glands were evaluated for RFP expression 1 day (D) and 1, 2 and 6 months (M) following TAM administration (left panel). Quantification of RFP^+^ cells per total number of cells (nuclei) counted in glands isolated at the indicated time points after TAM administration (right panel). (**D**) Co-staining of RFP and the proliferative marker Ki67 in adult submandibular glands 1 day after TAM administration (left panel). Quantification of RFP^+^/Ki67^+^ cells in submandibular glands, expressed as a percentage of total Ki67^+^ cells (right panel). Data are represented as mean ± standard deviation (S.D.). Arrows highlight double positive cells as indicated. D-day, M-month. Scale bar 37 μm. *n* = *4*.

Recent studies in the mammary and lacrimal gland have demonstrated that SMA^+^ cells function as lineage restricted progenitor cells in adult tissues, suggesting that SMA^+^ cells only contribute to the myoepithelial cell lineage^41,42^. To assess whether SMA^+^ cells in the submandibular gland function in a similar fashion, we performed genetic lineage tracing experiments in adult *Acta2*^*CreERT2*^*;Rosa26-tdTomato* (SMA bi-genic) mice. Six-week old SMA bi-genic animals were administered TAM and submandibular glands were obtained at various time points over a 6-month period (Fig. 7A). As expected, 6 months following TAM administration immunofluorescence analysis of submandibular glands revealed that the RFP^+^ cells were co-localized to the K14^+^ and ∆Np63^+^ cell populations, similar to our observation in the embryonic studies (Figs 5B, 7B, respectively). Indeed, the RFP^+^/K14^+^ and RFP^+^/∆Np63^+^ cells could be detected as early as 1 day post TAM administration (Supplementary Fig. S9). In contrast, we did not detect any RFP expression in the Nkcc1^+^ acinar cells of the glands suggesting a clear dichotomy of lineage contribution to this cellular population (Fig. 7B). Surprisingly, upon careful examination of the ducts, we identified a modest number of RFP^+^ cells that were also K7^+^ suggesting that SMA^+^ myoepithelial cells can contribute in part, to the ductal cell lineage (Fig. 7B). Indeed, these RFP^+^/K7^+^ ductal cells were detected as early as 1 month post TAM administration in both male and female animals (Supplementary Figs S9, S10 and S11).

Myoepithelial cells have long been thought to represent a differentiated cell population. To assess the replicative behaviors of the SMA^+^ myoepithelial cells we quantified the number of RFP^+^ cells in the SMA bi-genic mice 1 day and 1, 2 and 6 months post TAM administration. Our analysis revealed a modest increase in the number of RFP^+^ cells in the submandibular gland suggesting that the SMA^+^ myoepithelial cells may represent a long-lived cell population with the potential for some degree of self-renewal (Fig. 7C). We further assessed the proliferative properties of these cells. Quantification of 6-week old SMA bi-genic mice that were administered TAM, followed by a 1 day chase, showed that approximately 11% were RFP^+^/Ki67^+^ double positive and in active phases of the cell cycle, confirming active proliferation of MECs in adult submandibular glands (Fig. 7D). Overall, our genetic lineage tracing studies revealed that p63^+^ cells function as multipotent stem/progenitor cells contributing to and maintaining all epithelial cell lineages during both embryogenesis and in the adult gland. Conversely, while SMA^+^ MECs are multipotent, they only maintained the myoepithelial and ductal cell lineages in adults.

## Discussion

Unearthing the level of cellular heterogeneity within the salivary gland is critical for better understanding the biology of this organ during both normal physiological and diseased states. Using scRNA-seq we have generated a comprehensive map of the transcriptome of young adult mouse submandibular gland epithelial cells. While our scRNA-seq analysis confirmed the identity of the three major epithelial cell populations, additional hierarchical clustering revealed 7 clusters of epithelial cells. Our analysis highlights the remarkable level of heterogeneity within this gland, something which has not been previously reported for this organ, offering unprecedented insight into the epithelial lineage relationships within the gland. To better define the cellular hierarchy within this organ, we focused on the basal and myoepithelial subpopulation of cells. Using *in vivo* lineage tracing technologies, we mapped the cell fate of the basal and myoepithelial cells during development and in adults and have identified novel players in the salivary gland stem/progenitor cell hierarchy. Overall, our study has not only generated a large resource of single-cell gene expression profiles from murine salivary gland epithelial cells, but our *in vivo* study offers a major advancement in our understanding of salivary gland stem/progenitor cell biology.

Over the last several years a great amount of effort has been directed towards identifying stem/progenitor cells in the salivary gland. This has largely been driven by the importance of this cell population in various aspects of salivary gland biology including cell fate specification during morphogenesis, adult tissue regeneration and repair and the role of stem cells in carcinogenesis. Indeed, studies mapping cell fate trajectories have been instrumental in identifying central players in the salivary gland stem/progenitor cell hierarchy. Utilizing *in vivo* genetic lineage tracing technologies, we have identified two new markers that maintain the various mature cell lineages of the salivary gland. By irreversibly labeling p63^+^ cells and monitoring the cell fate trajectories of these cells, we find that p63^+^ cells function as multipotent stem/progenitor cells that contribute to and maintain all the epithelial cell lineages during organogenesis and in adult glands. In contrast to previous lineage tracing studies performed in the salivary gland, to our knowledge, this is the first report identifying a single stem/progenitor cell population that was capable of maintaining all of the mature epithelial cell lineages in the adult gland.

Recent studies tracing the progeny of K14^+^ cells in the salivary gland have reported that these cells maintain the ductal cell lineage^16^. Our analysis of the various subpopulations of ΔNp63^+^/K14^+^ cells revealed several unique cellular populations which were ΔNp63^+^/K14^−^, ΔNp63^−^/K14^+^ or ΔNp63^+^/K14^+^, highlighting the level of heterogeneity within the basal cells of the salivary gland (Supplementary Fig. S4A). These observations raised the possibility that various basal subpopulations contribute differently to normal gland maintenance with the p63^+^/K14^−^ stem/progenitor cells having a higher degree of stem/progenitor cell potential and subsequently maintain all epithelia cell lineages. In contrast, p63^+^/K14^+^ and/or p63^−^/K14^+^cells may function exclusively in maintaining the ductal cell lineage^16,17^. Interestingly, our results are in good agreement with similar studies performed in the prostate which have demonstrated that p63^+^ basal cells differentiate into all the epithelial cell lineages during development, in adult glands and glands undergoing regeneration^38,47^. Similar roles for p63 have also been reported in the epithelium of the respiratory and upper gastrointestinal tracts^45,48^.

Based on the spatial expression analysis of the p63 protein, it is clear that p63 is expressed in both the basal and myoepithelial cells. In order to better define the branch points in stem cell fate trajectories between the basal and myoepithelial cell populations, we performed lineage tracing analysis to map the cell fate of myoepithelial cells. Myoepithelial cells have long been thought to function primarily as the powerhouse for contractile forces required in the expulsion of secretory fluids in glandular organs such as the mammary, lacrimal and salivary gland. However, mounting evidence suggests a role for myoepithelial cells serving as a reservoir of stem/progenitor cells in mammary and lacrimal glands^41,42,49,50^. Indeed, our adult lineage tracing analysis of myoepithelial cells has identified a long-lived myoepithelial cell population which may function as multipotent stem/progenitor cells maintaining both the myoepithelial and ductal cell lineages. Although, these observations are in contrast to previous lineage tracing studies performed in the adult mammary and lacrimal glands which reported a lineage-restricted role by exclusively maintaining the myoepithelial cell population, our finds are supported by the scRNA-seq studies described in this report^41,42^. Our analysis of single-cell transcriptomes of the salivary gland uncovered a basal cluster (C6) of myoepithelial cells which expressed the myoepithelial specific genes *Acta2*, *Myh11* and *Myl9* in addition to ductal and acinar specific genes, which may represent a mixed-lineage, or an intermediate cell population (Fig. 1). While our scRNA-seq analysis focused on early postnatal day 8 (P8) glands, it is possible that the level of cellular heterogeneity in the myoepithelial cell population may display differences in adult glands. Indeed, a more systematic scRNA-seq analysis spanning different developmental stages and adults, would be valuable in both better understanding the degree of cell heterogeneity and in charting salivary gland hierarchical structure across developmental timepoints. Moreover, whereas we cannot definitively rule out caveats that are associated with scRNA-seq such as cell doublets or sequence library leakage^51^, it is tempting to speculate that these intermediate cells may represent a transient population of cells which are poised for commitment to the various cellular lineages, similar to what has been reported in the mammary gland^23,25^. Furthermore, our study highlights the level of cellular diversity and cell plasticity within the gland, properties which may be important for gland homeostasis or in response to injury or damage. Nevertheless, the identification of the cluster C6, which may represent a transient population of cells which express basal, ductal and acinar markers, and which may be poised to commit to the various cell lineages, supports the results of the *Acta2* lineage tracing studies and suggest that within the salivary gland epithelial cell hierarchy, there may exist a mixed-lineage population of cells which express *Acta2* and which are poised to commit to the ductal cell lineage.

**Figure 8:**
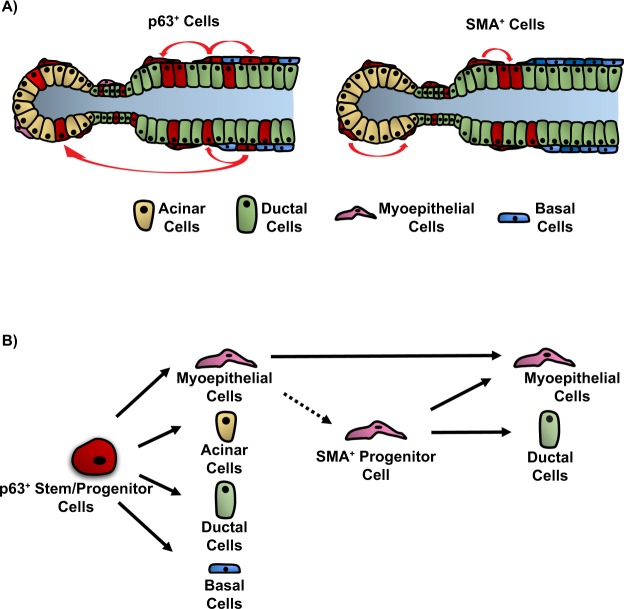
Model outlining the function of p63 and SMA stem/progenitor cells along the salivary gland epithelial cell hierarchy. (**A**) A schematic representation of the SMG. In adult glands, p63^+^ stem/progenitor cells maintain all the mature epithelial cell lineages (left panel). Conversely, SMA^+^ progenitor cells maintain the myoepithelial and ductal cell lineages in adult glands (right panel). (**B**) Proposed position of p63 and SMA in the salivary gland stem/progenitor cell hierarchy.

The identification of p63 and SMA as new players in the salivary gland cell hierarchy and understanding how they contribute to and maintain the mature cell lineages offer new insights into the intricate molecular networks that drive cell specification in this complex gland (Fig. 8). Further studies aimed at teasing out the signaling pathways and networks driving cell fate determination, lineage specification and differentiation programs in these stem/progenitor cell populations will aid in the development of new strategies for use in regeneration and tissue engineering approaches to treat patients with salivary gland dysfunction.

## Methods

### Animal experiments

All animal experiments and procedures were performed in accordance with the State University of New York at Buffalo (University at Buffalo) Institutional Animal Care and Use Committee (IACUC) regulations. All procedures were approved by University at Buffalo IACUC. Wild type C57BL/6J (Stock No. 000664) and *Rosa26-tdTomato* (B6.Cg-*Gt(ROSA)26Sor*^*tm14(CAG-tdTomato)Hze*^/J; Stock No. 007914) mice were purchased from The Jackson Laboratory (Bar Harbor, Maine). The *Acta2*^*CreERT2*^ mice were a generous gift from Pierre Chambon and have been previously described^40^. Establishment of the *Trp63*^*CreERT2*^ mice have been previously reported^38^. For timed pregnancies, indicated mice were mated and noon of the day the vaginal plug was observed was considered E0.5. Animals were euthanized by CO_2_ inhalation and submandibular glands were dissected from animals at specific embryonic and adult time points.

### Lineage tracing experiments

For lineage tracing experiments performed during embryogenesis, indicated mice were crossed with *Rosa26-tdTomato* animals and 2 mg of the inactive form of tamoxifen (Sigma Cat.# T-5648) dissolved in corn oil was administered to the embryos by intraperitoneal injection to pregnant females. Embryos were harvested at E18.5. For adult studies, 6-week old adult *Acta2*^*CreERT2*^*; Rosa26-tdTomato* or adult *Trp63*^*CreERT2*^*; Rosa26-tdTomato* mice received one injection of 2 mg of tamoxifen dissolved in corn oil by intraperitoneal injection and a second intraperitoneal injection of 2 mg of tamoxifen in corn oil was administered three days later. Control mice received corn oil injections only. Mice were euthanized by CO_2_ inhalation and submandibular glands were harvested 1 day, 1 week, 1 month, 2 months, and 6 months post-injection/labeling.

### Single-cell RNA sequencing

Single cell suspensions from freshly isolated P8 murine submandibular glands were generated as previously described^52^. Submandibular gland cellular suspensions (~10,000 cells) were loaded on a Chromium Single Cell Instrument (10X Genomics) to generate single-cell gel bead-in-emulsions (GEMs). Single-cell RNA-seq libraries were prepared using Chromium Single cell 3′ Reagent Kits v2 Library (10X Genomics). Libraries were run using paired end sequencing on Illumina HigSeq2500 using the following cycles: Read 1–26 cycles, i7 index-8 cycles, i5 index: 0 cycles and Read 2: 110 cycles. The Cell Ranger Suite version 2.0.2 software was used to perform sample barcode processing and single-cell gene UMI (unique molecular index) counting (http://software.10xgenomics.com/single-cell/overview/welcome). Reads were aligned to the mm10 reference genome using the pre-built annotation package provided on the 10X Genomics website. A total of 1,013 cells were sequenced to a depth of 301,852 mean reads per cell and 2,241 median genes per cell. Only confidently mapped, non-duplicate with valid barcodes and UMIs were used to generate a gene-barcode matrix for further analysis. Data were analyzed using R and the R package Seurat for single cell analysis. Basic filtering was used to discard cells with fewer that 200 genes expressed and to remove genes expressed in fewer than 3 cells. A complete list of differentially expressed genes are provided in Supplementary Table S1.

### Immunostaining and imaging

Paraffin embedded submandibular gland tissue sections were process for immunofluorescent analysis as previously described^53^. Primary antibodies used at the indicated dilutions include alpha-smooth muscle actin (SMA) (1:200, Sigma, 1A4), K7 (1:50, Abcam), K14 (1:100^53^), K5 (1:100, gift from Dr. Julie Segre), Ki67 (1:100, Leica Biosystems, MM1), Nkcc1 (1:100, Santa Cruz Biotechnology), ΔNp63 (1:25)^54^, p63 (1:25, Biocare Medical, 4A4), p63 (1:25, Cell Signaling Technology, D2K8X), RFP (1:100, Rockland), DsRed (1:50, Clontech), Mist1 (1:100, Abcam), Aqp5 (1:100, Alomone Labs). Sections were mounted using VECTASHIELD Antifade Mounting Medium with DAPI (Vector Laboratories) and imaged using a ZEISS Confocal microscope with ZEISS ZEN imaging software.

### Quantification analyses

All analyses were performed using confocal images and quantified using ImageJ (NIH; Bethesda, Maryland). Quantifications are represented in Supplementary Table S2.

### Analysis of cell proliferation

The percentage of RFP^+^/Ki67^+^ double positive cells were calculated by counting the total number of RFP^+^/Ki67^+^ double positive cells divided by the total number of Ki67^+^ cells. A total of 10 fields of view (400x) were used for each quantification analysis. Approximately 350 ± 50 nuclei were counted per field and a total of ~4,000 ± 300 nuclei were counted per animal. Values are reported as mean ± standard deviation (S.D.). *n* = *4*.

### Quantification of total RFP^+^ cells

The percentage of RFP positive cells was calculated by counting the total number of RFP^+^ positive cells divided by the total number of nuclei. A minimum of 10 fields of view (400x) were used for each quantification analysis. A total of ~3,700 ± 800 nuclei were counted per animal. Values are reported as mean ± standard deviation (S.D.). *n* = 4.

### Quantification of the number of RFP^+^ cells that co-express various cell lineage markers

The percentage of RFP^+^ cells that co-express a cell lineage marker (K14, SMA, p63, K7, Nkcc1, Mist1, Aqp5) were calculated by counting the number of double positive RFP and lineage specific marker cells, divided by the total number of RFP^+^ cells. The SMA^+^/K14^+^/RFP^+^ triple positive cells and the SMA^−^/K14^+^/RFP^+^ double positive cells were quantified and divided by the total number of RFP^+^ cells to calculate the percentage of RFP^+^ myoepithelial cells and RFP^+^ basal cells respectively during embryogenesis. Values are reported as mean ± standard deviation (S.D.). *n* = 4.

### Evaluation of co-expression of basal and myoepithelial cell markers

Embryonic day E18.5 and 8-week old wild type C57BL/6J submandibular glands were stained with various combinations of SMA, K5, K14, and ∆Np63. The percentage of single positive SMA^+^, single positive K14^+^, or double positive SMA^+^/K14^+^ cells were calculated by dividing each cell population of interest, by the total number of SMA^+^, K14^+^, and SMA^+^/K14^+^ cells. All cellular populations evaluated were calculated in a similar fashion. A minimum of 10 fields of view (400x) were used for each quantification analysis. A total of 3,400 ± 400 nuclei were counted per animal. Values are reported as mean ± standard deviation (S.D.). *n* = *3*.

### Statistical analysis

Quantified results were reported as mean ± standard deviation (S.D.) of three or more independent experiments. All quantifications are represented in Supplementary Table S2.

## Electronic supplementary material

Supplementary Information

Supplementary Table S1

Supplementary Table S2

## Data Availability

Sequencing data has been deposited in the Gene Expression Omnibus (GEO) database under the accession number GSE113466.
