# Supplementary material for: Genetic and scRNA-seq Analysis Reveals Distinct Cell Populations that Contribute to Salivary Gland Development and Maintenance
Source: Sci Rep. 2018 Sep 19;8:14043. doi: 10.1038/s41598-018-32343-z (PMC6145895; doi:10.1038/s41598-018-32343-z)
Supplement: Supplementary file 1 — Supplementary Information [file 41598_2018_32343_MOESM1_ESM.pdf]

Genetic and scRNA-seq Analysis Reveals Distinct Cell Populations that Contribute to Salivary Gland Development and Maintenance

Eun-Ah Christine Song[#1], Sangwon Min[#1], Akinsola Oyelakin[1], Kirsten Smalley[2], Jonathan E Bard[3], Lan Liao[4], Jianming Xu[4], and Rose-Anne Romano[1,2]*

[1]Department of Oral Biology, School of Dental Medicine, State University of New York at Buffalo, Buffalo, New York, 14214, USA.
[2]Department of Biochemistry, Jacobs School of Medicine and Biomedical Sciences, State University of New York at Buffalo, Buffalo, New York, 14203, USA.
[3]Genomics and Bioinformatics Core, State University of New York at Buffalo, Buffalo, New York 14222, USA.
[4]Department of Molecular and Cellular Biology, Baylor College of Medicine, Houston, Texas, 77030, USA.

# Authors contributed equally to this work

* To whom correspondence should be addressed: rromano2@buffalo.edu

Figure S1. Expression analysis of p63 and SMA based on transcriptomic profiling by RNA-sequencing of mouse submandibular glands during development and in adults. (**A**) Snapshot from the University of California, Santa Cruz (UCSC) genome browser showing distribution of the aligned RNA-seq reads at the *Trp63* genomic locus in mouse submandibular glands during development and in adults. (**B-C**) *Trp63* and *Acta2* mRNA expression levels were plotted as Fragments Per Kilobase of transcripts per Million mapped reads (FPKM) based on RNA-sequencing studies performed in the submandibular gland at various developmental stages and in adults[36]. Data are represented as mean ± standard deviation (S.D.). Shaded grey boxes highlight exon 1 of the TAp63 genomic locus and the unique exon of the ΔNp63 locus. E- embryonic, P- postnatal.

Figure S2. Expression and quantification of ΔNp63, K5, and K14 in embryonic salivary glands at E18.5. (**A**) Expression pattern of ΔNp63 and K5 in E18.5 salivary glands. Single channel and merged images are shown. Arrow- $\Delta Np63^+/K5^+$ cells, white arrowhead- $\Delta Np63^-/K5^+$ cells, orange arrowhead- $\Delta Np63^+/K5^-$ cells. (**B**) Expression of ΔNp63 and K14 in E18.5 glands. Single channel and merged images are shown. Arrow- $\Delta Np63^+/K14^+$ cells, white arrowhead- $\Delta Np63^-/K14^+$ cells, orange arrowhead- $\Delta Np63^+/K14^-$ cells. (**C**) Quantification of $\Delta Np63^+$ and $K5^+$ cell populations in E18.5 glands. (**D**) Quantification of $\Delta Np63^+$ and $K14^+$ cell populations in E18.5 glands. Data are represented as mean ± standard deviation (S.D.). Scale bar 37μm. *n=3.*

Figure S3. Spatial expression analysis of p63 and SMA in adult glands. Eight-week old adult submandibular glands were evaluated for expression of ΔNp63 and SMA, together with Nkcc1. Scale bar 37μm.

Figure S4. Expression and quantification of ΔNp63, K5 and K14 in adult submandibular glands. (**A**) Left panel demonstrates the expression of ΔNp63 and K14 in 8-week adult submandibular salivary glands. Quantification of ΔNp63^{+} and K14^{+} cell populations (right panel). Arrow- ΔNp63^{+}/K14^{+} cells, white arrowhead- ΔNp63^{-}/K14^{+} cells, orange arrowhead- ΔNp63^{+}/K14^{-} cells. (**B**) Expression of ΔNp63 and K5 in 8-week adult submandibular salivary glands (left panel). Quantification of ΔNp63^{+} and K5^{+} cell populations (right panel). Arrow- ΔNp63^{+}/K5^{+} cells, white arrowhead- ΔNp63^{-}/K5^{+} cells, orange arrowhead- ΔNp63^{+}/K5^{-} cells. Data are represented as mean ± standard deviation (S.D.). Scale bar 37μm. *n=3.*

Figure S5. Co-localization of RFP expression with ΔNp63 and SMA in adult mouse submandibular glands. (**A**) TAM was administered to 6-week old adult *Trp63^{CreERT2};Rosa26-tdTomato* (p63 bi-genic) mice and glands were harvested after 1 week. Co-staining with RFP and ΔNp63 revealed faithful expression of RFP and ΔNp63. (**B**) Similar experiments performed in *Acta2^{CreERT2};Rosa26-tdTomato* (SMA bi-genic) animals after 1 day of TAM administration showed faithful expression of RFP and SMA. Scale bar 37μm.

Figure S6. Contribution of SMA^{+} cells during salivary gland development. (**A**) Genetic lineage tracing studies performed in the *Acta2^{CreERT2};Rosa26-tdTomato* mice. RFP expression was induced at E16.5 by injection of TAM to pregnant females and cells were traced for 2 days and glands were dissected at E18.5. Glands were stained for RFP/K14/SMA expression (left panel). Quantification of the percent RFP^{+}, SMA^{+}, K14^{+} cells is shown in right panel. (**B**) Single channel images are shown. Arrow- RFP^{+}/K14^{+}/SMA^{+} cells, white arrowhead- RFP^{+}/K14^{+}/SMA^{-} cells, orange arrowhead- RFP^{-}/K14^{+}/SMA^{-} cells. Data are represented as mean ± standard deviation (S.D.). Scale bar 75μm. *n=4.*

Figure S7. Contribution of p63^{+} stem/progenitor cells in adult submandibular glands. (**A-B**) Outline of the experimental timeline used for the adult genetic lineage tracing experiments in *Trp63^{CreERT2};Rosa26-tdTomato* mice. Co-staining of RFP with the various mature epithelial cell lineage markers as indicated. Arrows indicate co-localization of RFP^{+} cells with the various cell lineage markers as indicated. Scale bar 37μm.

Figure S8. p63^{+} stem/progenitor cells maintain the acinar cell lineage. Z-stack images of male and female *Trp63^{CreERT2};Rosa26-tdTomato* submandibular glands 6 months following TAM administration show co-localization of RFP with the acinar cell markers Aquaporin 5 (Aqp5), Nkcc1, and Mist1 as indicated (upper panel). Lower panel shows quantification of the percentage of RFP^{+}/Aqp5^{+}, RFP^{+}/Nkcc1^{+}, and RFP^{+}/Mist1^{+}

cells. Arrows indicate co-localization of RFP^+ cells with the various acinar cell lineage markers. Scale bar 37μm. *n*=4.

Figure S9. Contribution of SMA^+ stem/progenitor cells in adult submandibular glands. (**A-B**) Outline of the experimental timeline used for the adult genetic lineage tracing experiments in *Acta2^{CreERT2};Rosa26-tdTomato* mice. Co-staining of RFP with markers for the various mature epithelial cell lineages as indicated. Arrows indicate co-localization of RFP^+ cells with the various cell lineage markers as indicated. Scale bar 37μm.

Figure S10. SMA^+ myoepithelial cells contribute to the $K7^+$ ductal cell lineage. Z-stack images of male and female *Acta2^{CreERT2};Rosa26-tdTomato* submandibular glands 1 month and 6 months following TAM administration show co-localization of RFP with the ductal cell marker K7. Arrows indicate $RFP^+/K7^+$ cells. Scale bar 37μm.

Figure S11. SMA^+ cells contribute to the $K7^+$ ductal cell lineage. Single channel, merged, and z-stacked images of male and female *Acta2^{CreERT2};Rosa26-tdTomato* submandibular glands 1 month and 6 months following TAM administration. Arrows indicate $RFP^+/K7^+$ cells. Scale bar 37μm.

A)

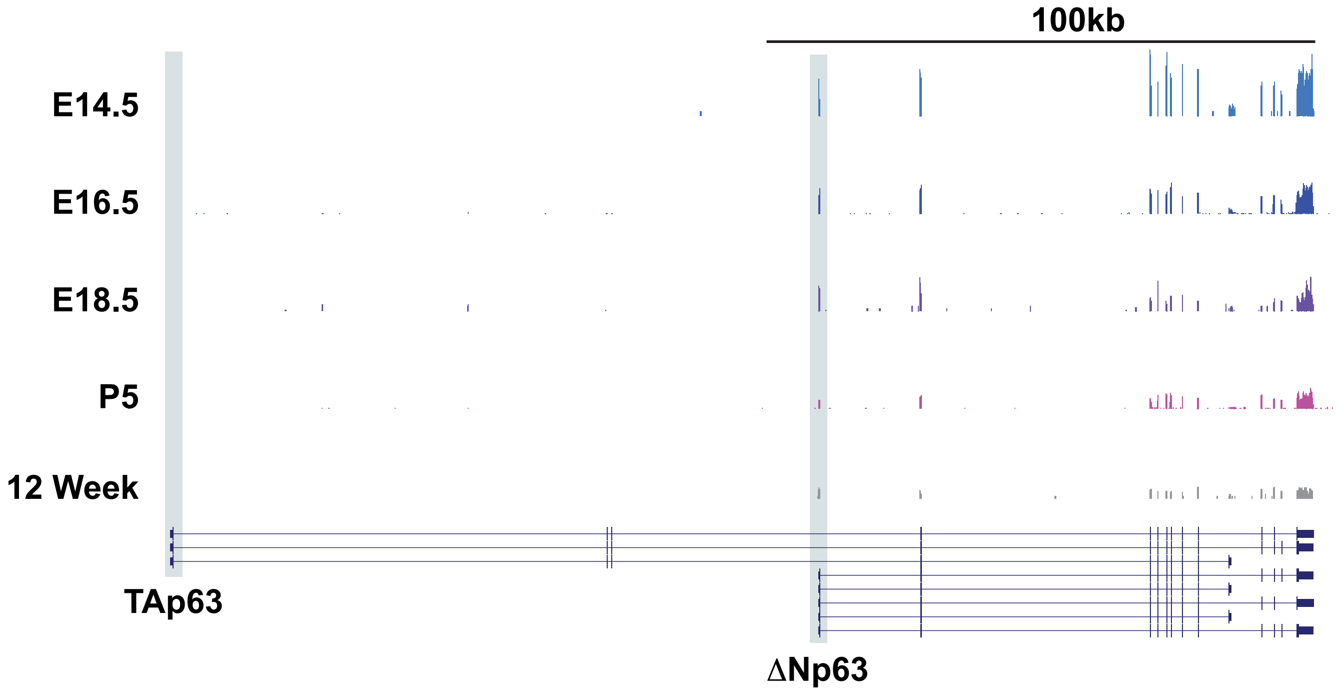

B)

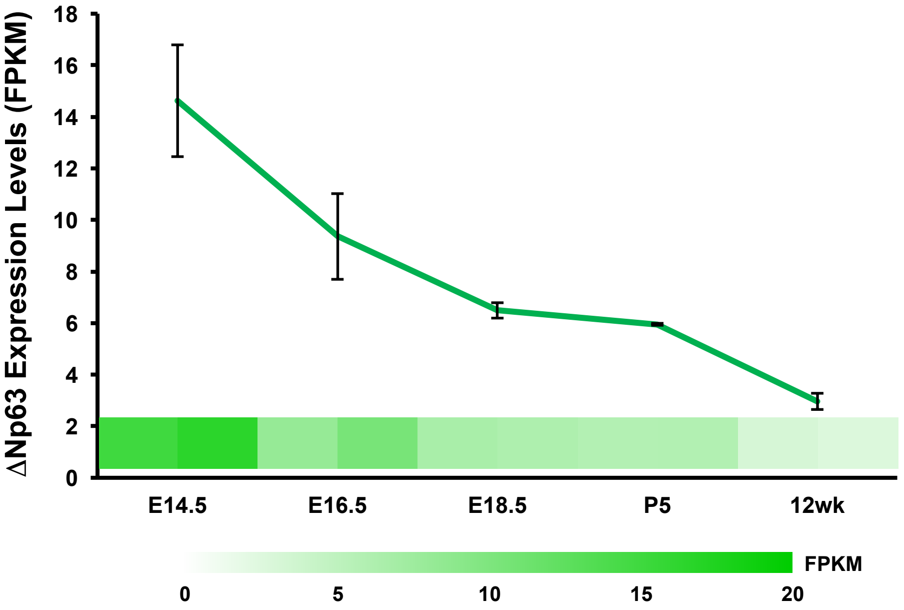

C)

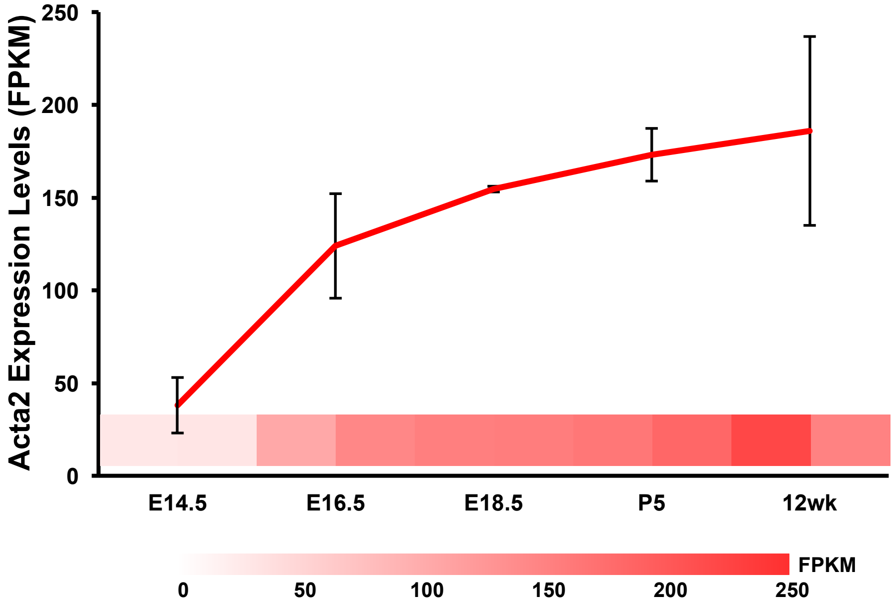

Figure S1.

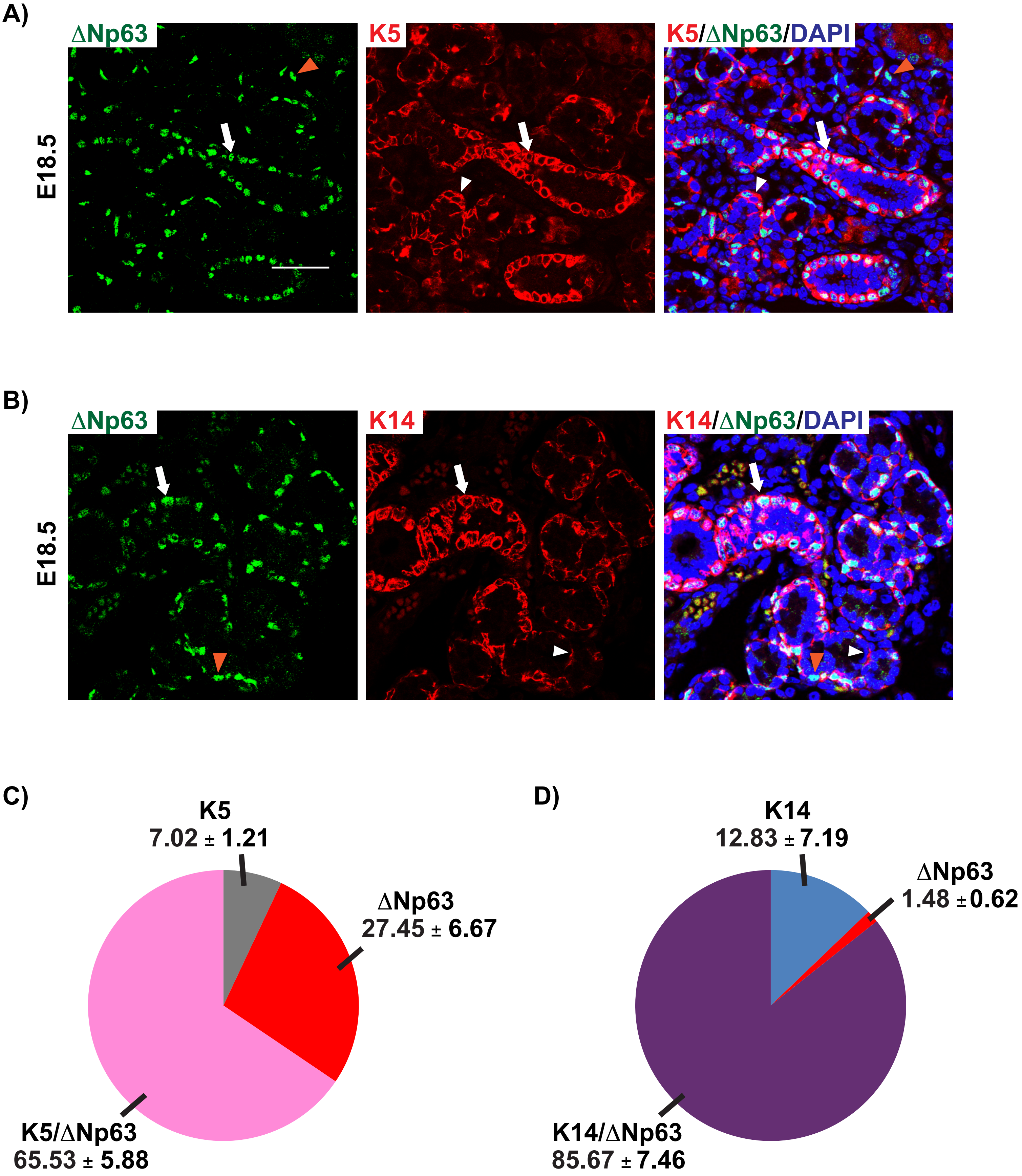
A)
ΔNp63
K5
K5/ΔNp63/DAPI
E18.5
B)
ΔNp63
K14
K14/ΔNp63/DAPI
E18.5
C)
K5
7.02 ± 1.21
ΔNp63
27.45 ± 6.67
K5/ΔNp63
65.53 ± 5.88
D)
K14
12.83 ± 7.19
ΔNp63
1.48 ± 0.62
K14/ΔNp63
85.67 ± 7.46

Figure S2.

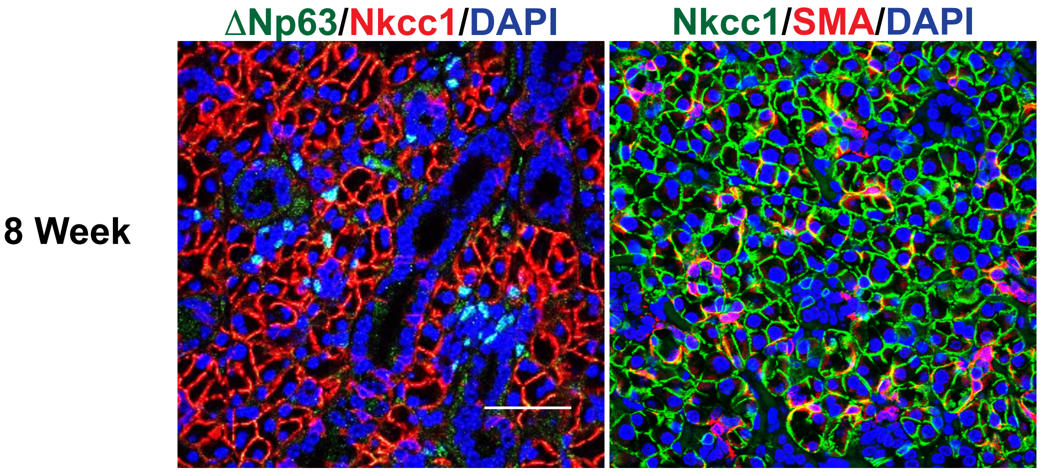

Figure S3.

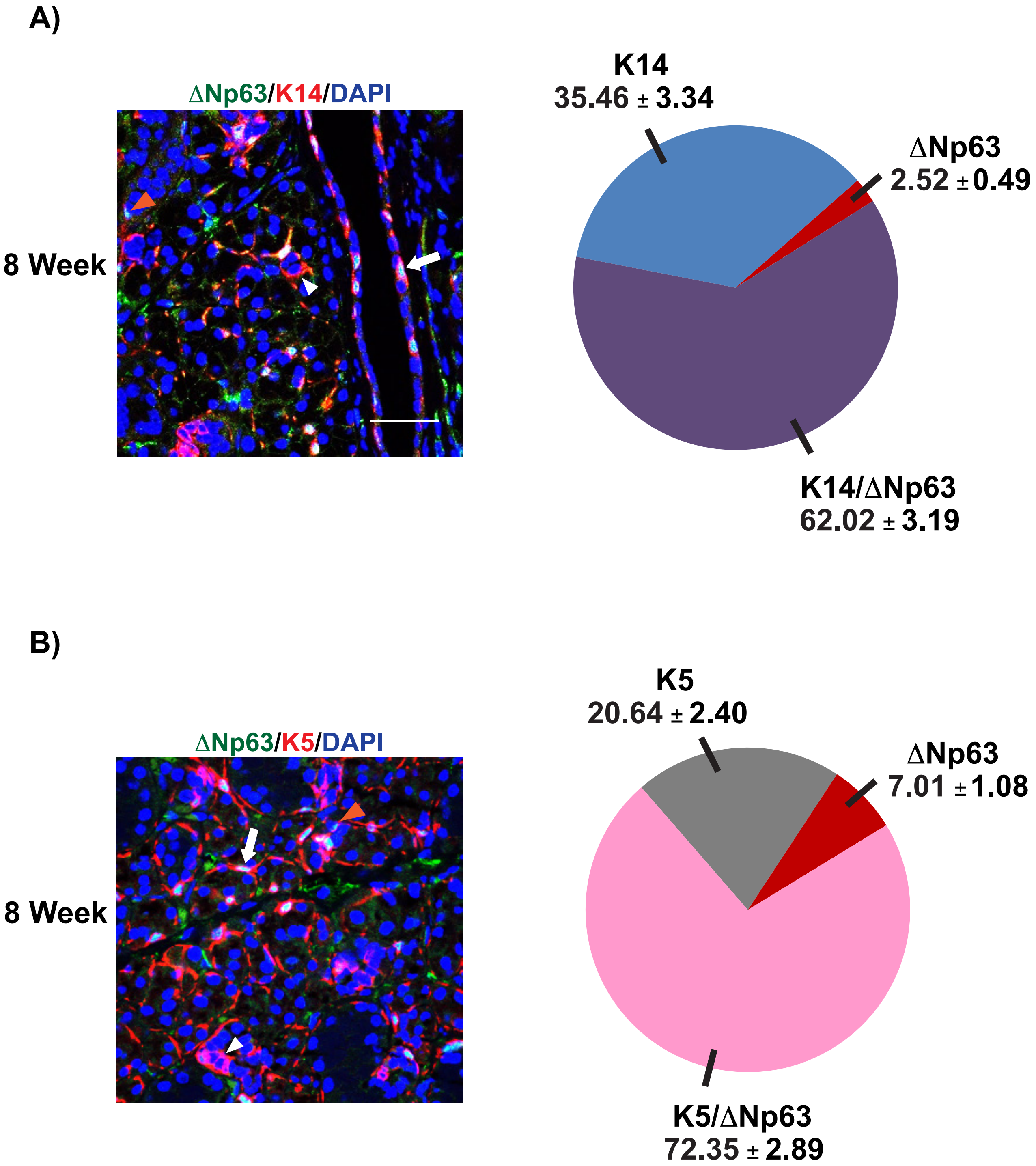
A)
ΔNp63/K14/DAPI
8 Week
K14
35.46 ± 3.34
ΔNp63
2.52 ± 0.49
K14/ΔNp63
62.02 ± 3.19
B)
ΔNp63/K5/DAPI
8 Week
K5
20.64 ± 2.40
ΔNp63
7.01 ± 1.08
K5/ΔNp63
72.35 ± 2.89

Figure S4.

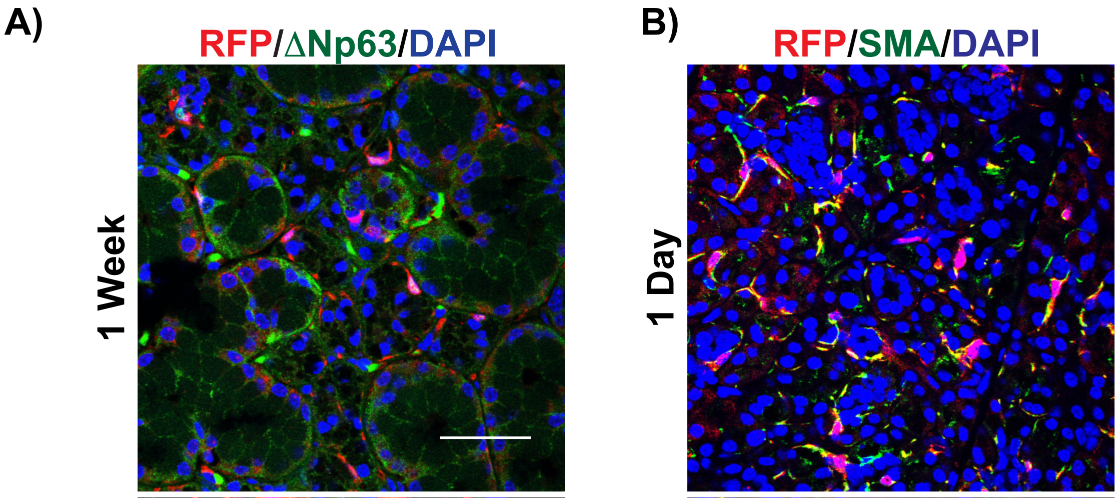

Figure S5.

A)

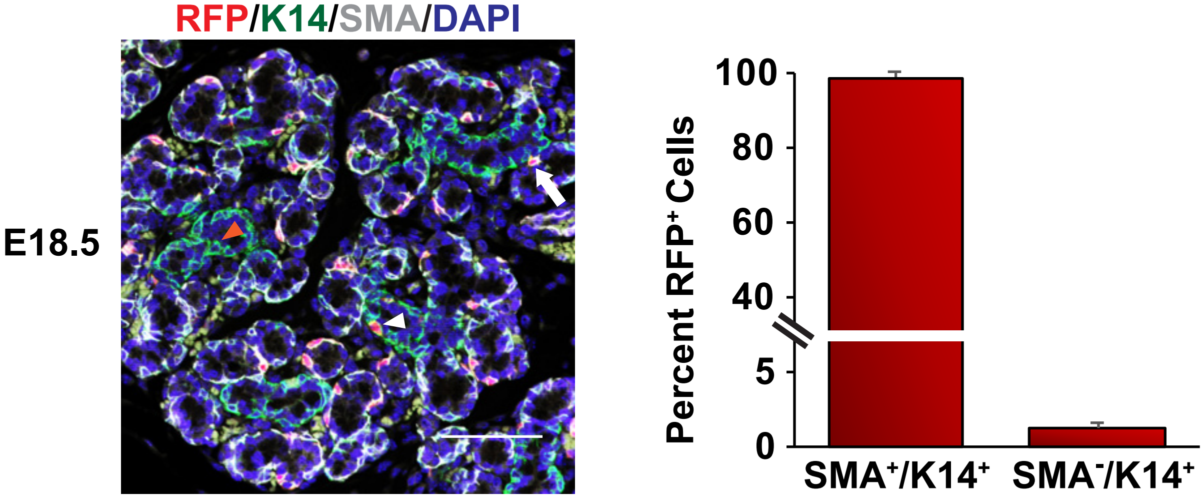
RFP/K14/SMA/DAPI
E18.5
Percent RFP+ Cells
100
80
60
40
5
0
SMA+/K14+
SMA-/K14+

B)

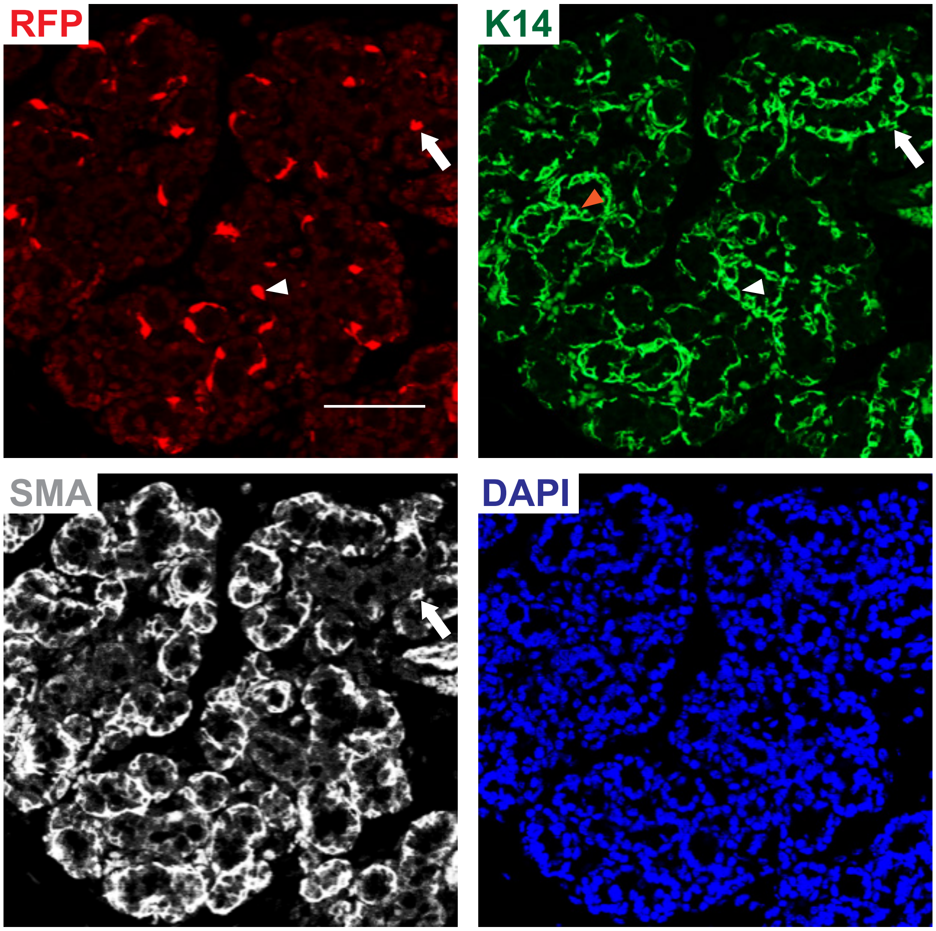
RFP
K14
SMA
DAPI

Figure S6.

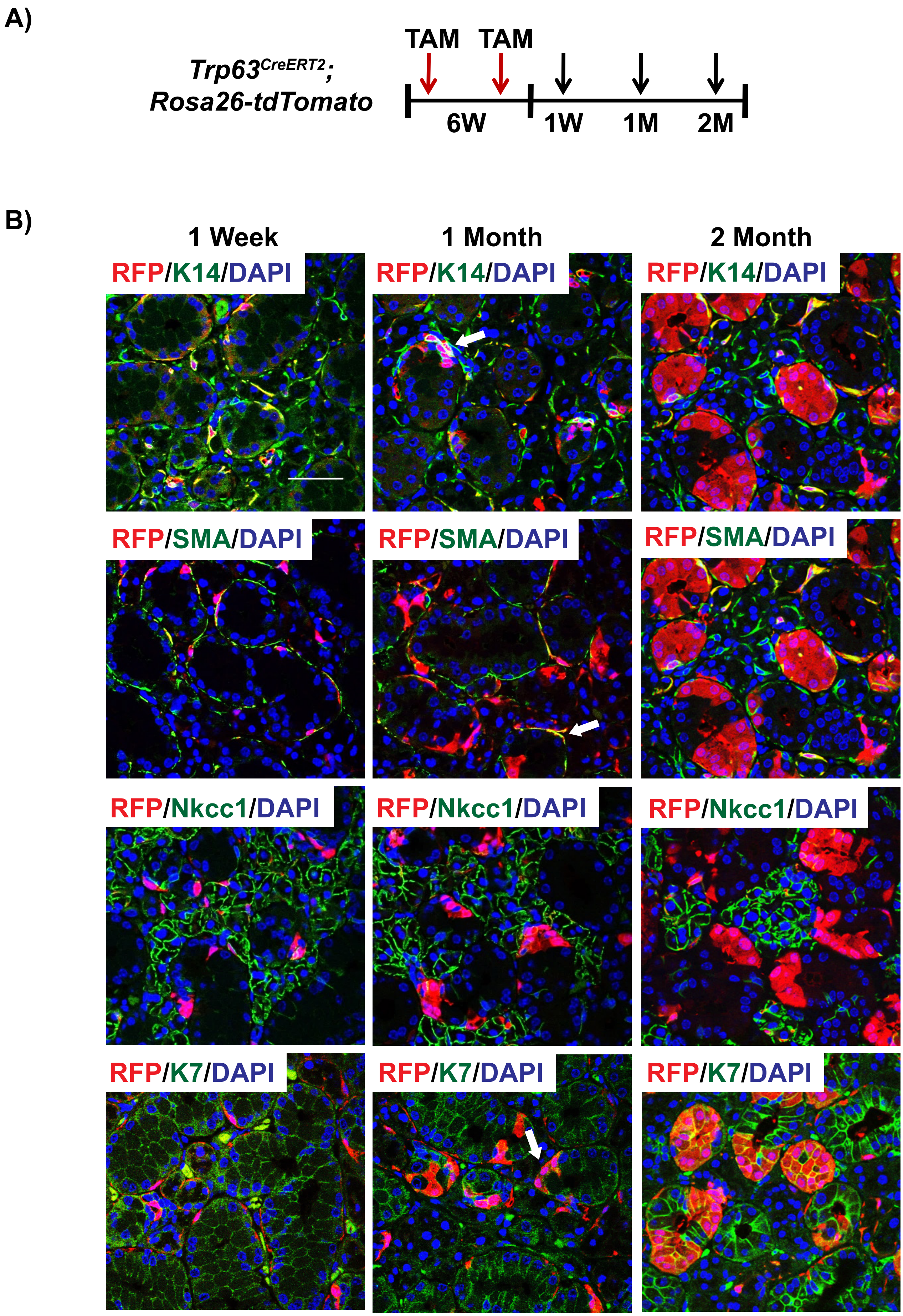
A)
Trp63CreERT2;
Rosa26-tdTomato
TAM
TAM
6W
1W
1M
2M
B)
1 Week
1 Month
2 Month
RFP/K14/DAPI
RFP/K14/DAPI
RFP/K14/DAPI
RFP/SMA/DAPI
RFP/SMA/DAPI
RFP/SMA/DAPI
RFP/Nkcc1/DAPI
RFP/Nkcc1/DAPI
RFP/Nkcc1/DAPI
RFP/K7/DAPI
RFP/K7/DAPI
RFP/K7/DAPI

Figure S7.

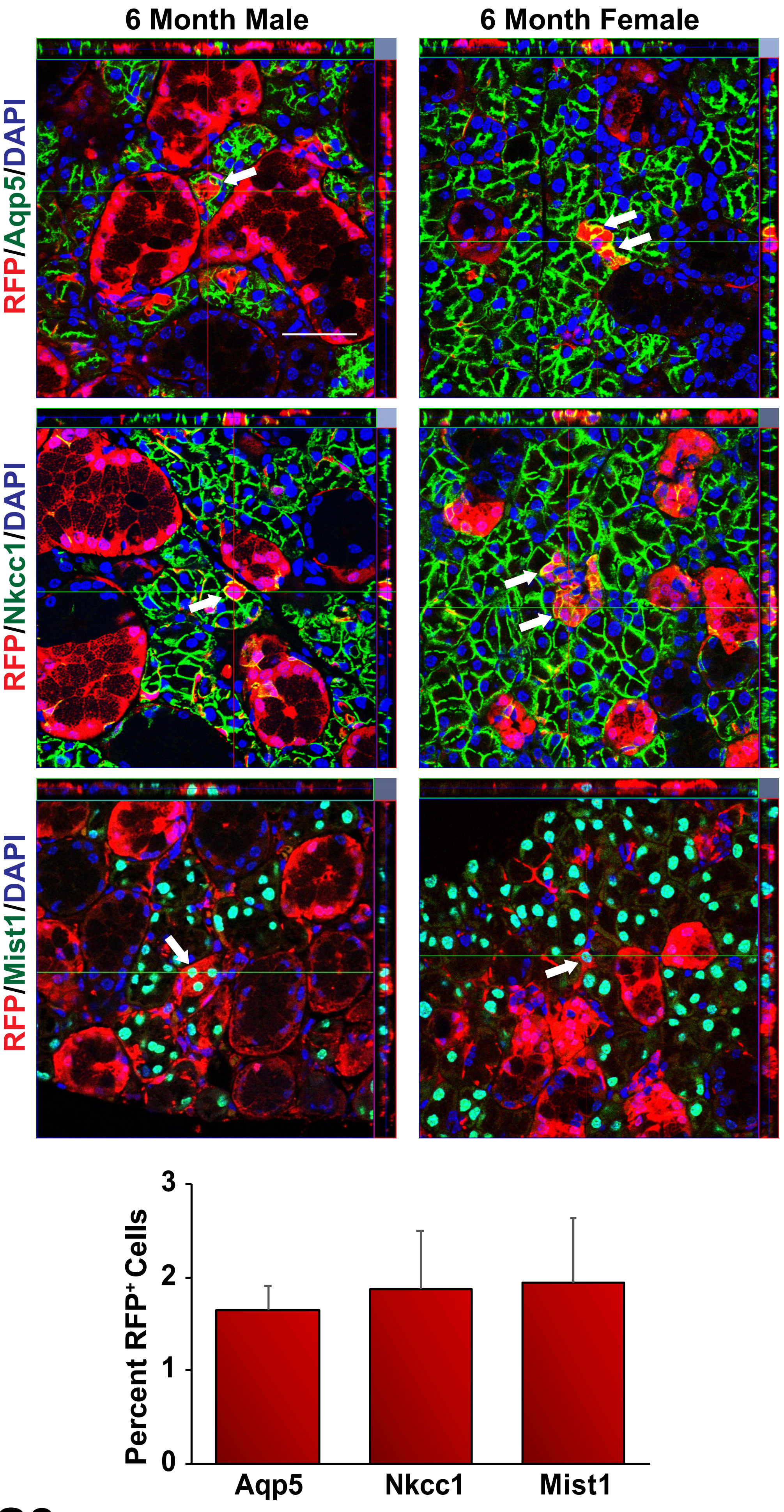
6 Month Male
6 Month Female
RFP/Aqp5/DAPI
RFP/Nkcc1/DAPI
RFP/Mist1/DAPI
Percent RFP⁺ Cells
3
2
1
0
Aqp5
Nkcc1
Mist1

Figure S8.

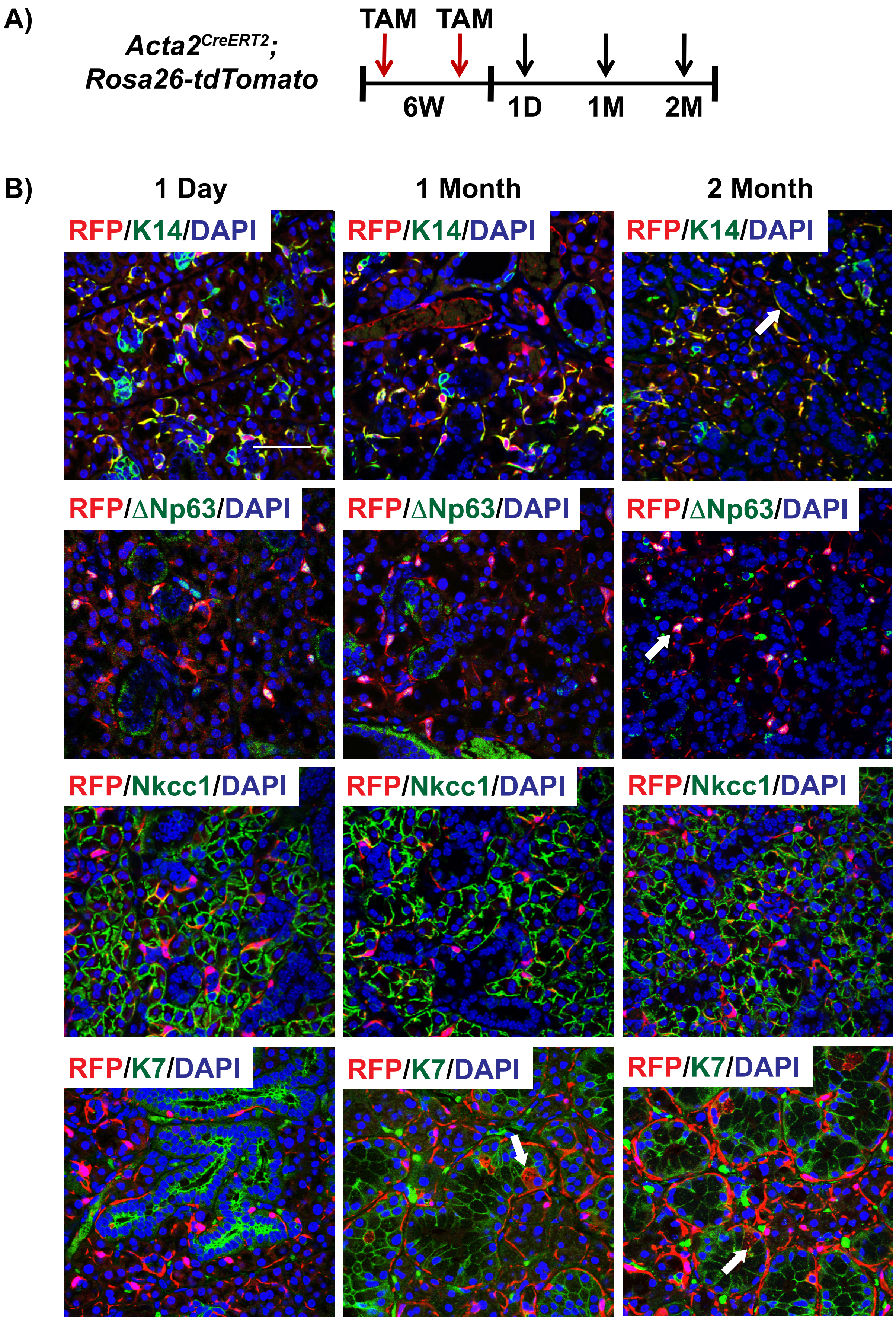
A)
Acta2CreERT2;
Rosa26-tdTomato
TAM
TAM
6W
1D
1M
2M
B)
1 Day
1 Month
2 Month
RFP/K14/DAPI
RFP/K14/DAPI
RFP/K14/DAPI
RFP/ΔNp63/DAPI
RFP/ΔNp63/DAPI
RFP/ΔNp63/DAPI
RFP/Nkcc1/DAPI
RFP/Nkcc1/DAPI
RFP/Nkcc1/DAPI
RFP/K7/DAPI
RFP/K7/DAPI
RFP/K7/DAPI

Figure S9.

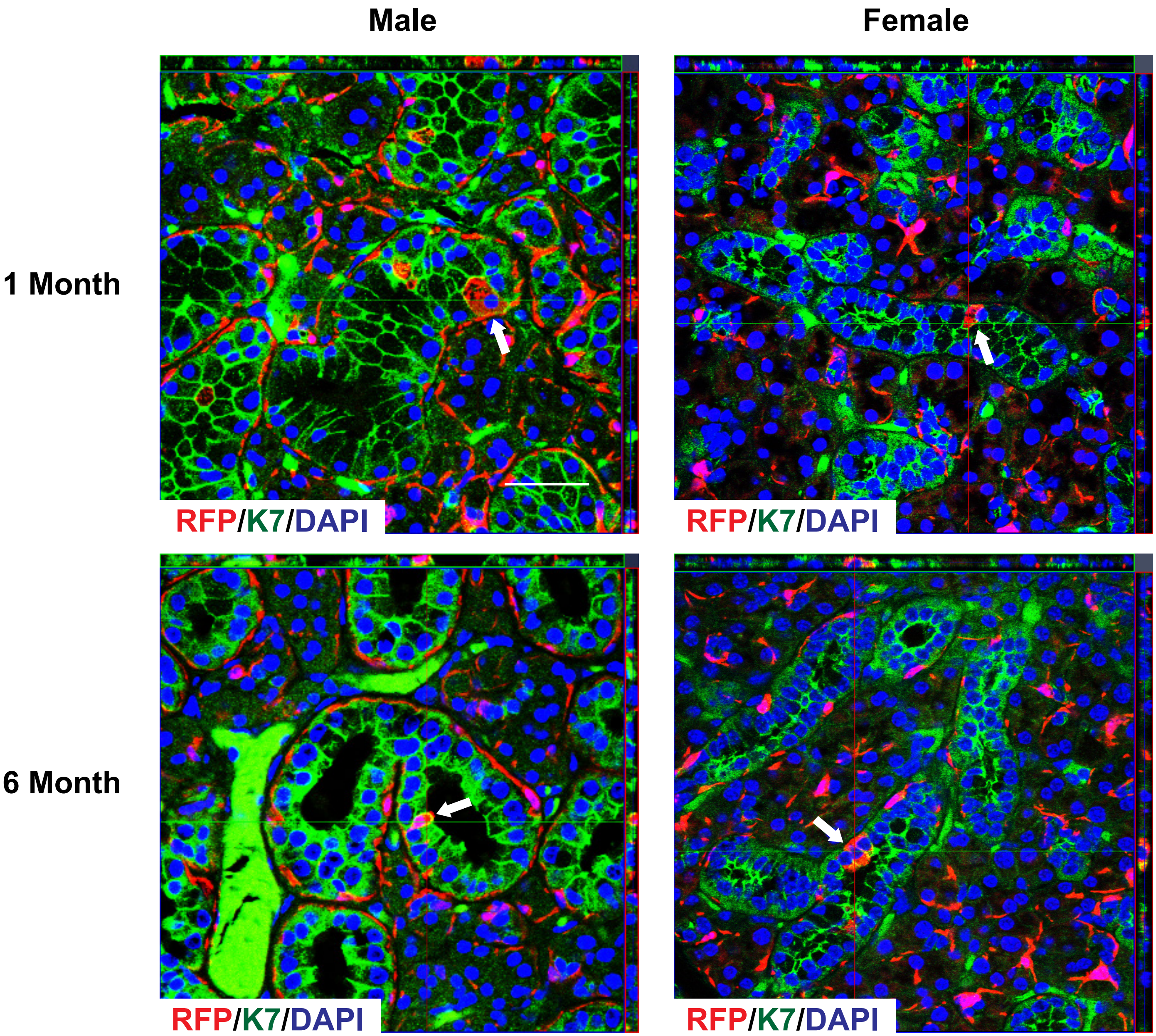
Male
Female
1 Month
6 Month
RFP/K7/DAPI
RFP/K7/DAPI
RFP/K7/DAPI
RFP/K7/DAPI

Figure S10.

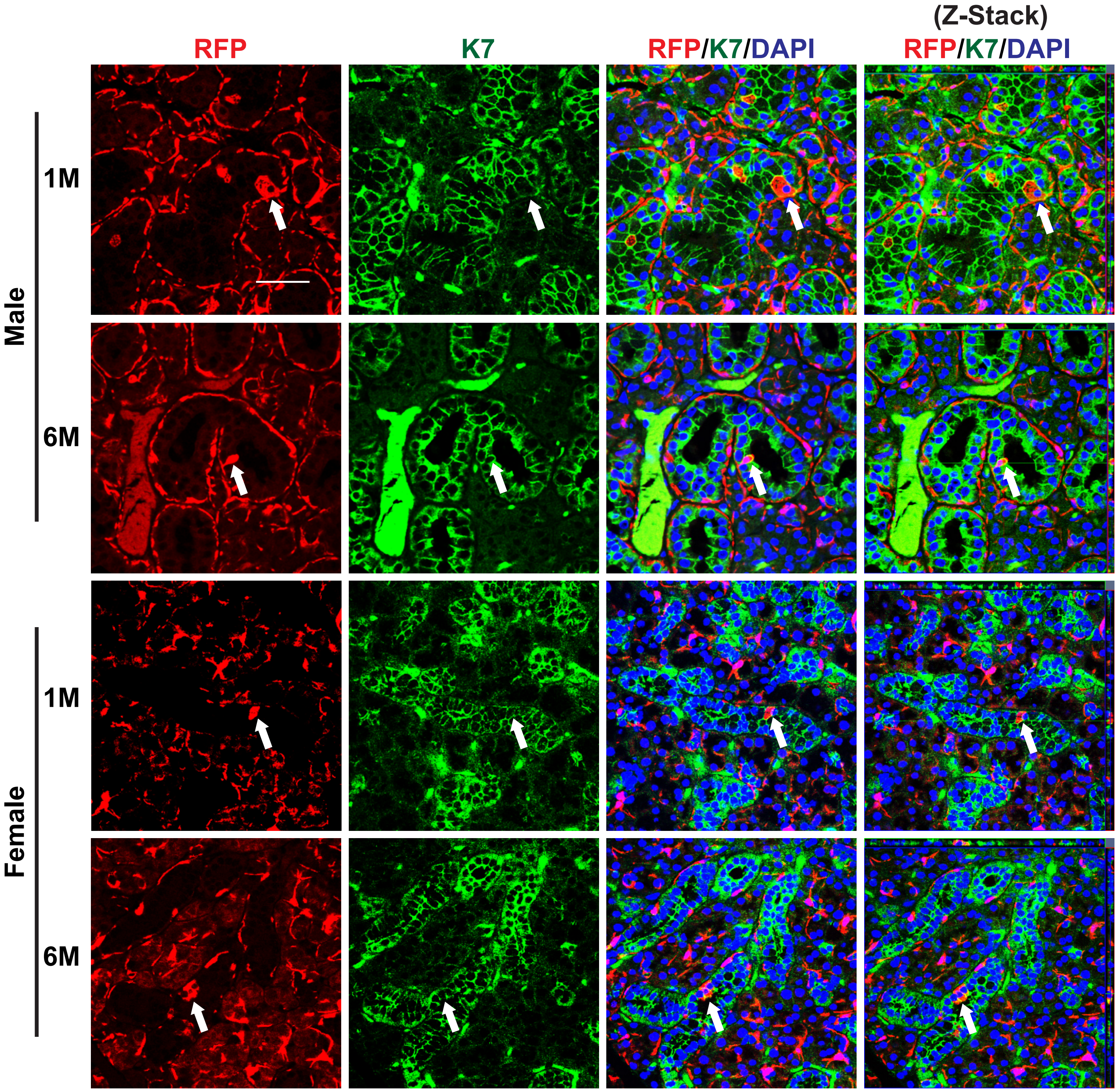

Figure S11.
